# Bog ecosystems as a playground for plant–microbe coevolution: bryophytes and vascular plants harbour functionally adapted bacteria

**DOI:** 10.1186/s40168-021-01117-7

**Published:** 2021-08-11

**Authors:** Wisnu Adi Wicaksono, Tomislav Cernava, Christian Berg, Gabriele Berg

**Affiliations:** 1 Institute of Environmental Biotechnology, Graz University of Technology, Graz, Austria; 2 Institute of Plant Sciences, University of Graz, Graz, Austria; 3 Leibniz Institute for Agricultural Engineering and Bioeconomy (ATB), Potsdam, Germany; 4 Institute for Biochemistry and Biology, University of Postdam, Postdam, Germany

**Keywords:** Bog ecosystems, Bryophytes, Vascular plants, Microbiome, Coevolution

## Abstract

**Background:**

Bogs are unique ecosystems inhabited by distinctive, coevolved assemblages of organisms, which play a global role for carbon storage, climate stability, water quality and biodiversity. To understand ecology and plant–microbe co-occurrence in bogs, we selected 12 representative species of bryophytes and vascular plants and subjected them to a shotgun metagenomic sequencing approach. We explored specific plant–microbe associations as well as functional implications of the respective communities on their host plants and the bog ecosystem.

**Results:**

Microbial communities were shown to be functionally adapted to their plant hosts; a higher colonization specificity was found for vascular plants. Bryophytes that commonly constitute the predominant *Sphagnum* layer in bogs were characterized by a higher bacterial richness and diversity. Each plant group showed an enrichment of distinct phylogenetic and functional bacterial lineages. Detailed analyses of the metabolic potential of 28 metagenome-assembled genomes (MAGs) supported the observed functional specification of prevalent bacteria. We found that novel lineages of *Betaproteobacteria* and *Actinobacteria* in the bog environment harboured genes required for carbon fixation via RuBisCo. Interestingly, several of the highly abundant bacteria in both plant types harboured pathogenicity potential and carried similar virulence factors as found with corresponding human pathogens.

**Conclusions:**

The unexpectedly high specificity of the plant microbiota reflects intimate plant–microbe interactions and coevolution in bog environments. We assume that the detected pathogenicity factors might be involved in coevolution processes, but the finding also reinforces the role of the natural plant microbiota as a potential reservoir for human pathogens. Overall, the study demonstrates how plant–microbe assemblages can ensure stability, functioning and ecosystem health in bogs. It also highlights the role of bog ecosystems as a playground for plant–microbe coevolution.

**Video abstract**

**Supplementary Information:**

The online version contains supplementary material available at 10.1186/s40168-021-01117-7.

## Introduction

Bog ecosystems are one of the oldest vegetation forms in the world; they play a central role in the global carbon cycle and storage as well as a source for fresh water [1–3]. This widespread ecosystem is commonly dominated by *Sphagnum* mosses due to their unique biochemical and morphological adaptation to acidic, water-saturated and nutrient-poor environments [4]. As a keystone species group, *Sphagnum* mosses engineer this ecosystem, i.e. by acidification due to cation exchange and by forming a deep anaerobic peat layer. Well-adapted, non-vascular (acidophytic liverworts and mosses) and vascular plants (graminoids and dwarf shrubs) commonly co-occur in low abundances embedded in the *Sphagnum* layer [5–7]; therefore, they have different strategies regarding their growth form and root architecture [6]. Interactions between *Sphagnum* mosses and the other plants are important for the formation of the hummock topology and nutrient cycling, which facilitates their growth in the bog ecosystem [5, 7]. The bog microbiome plays a crucial role for bog ecosystem functioning, nutrient cycling and protection towards biotic and abiotic stress [8–10]. Previously, a core microbiome or meta-community of bog plants was identified [11], but knowledge about plant-specific distribution patterns and functions is still missing.

Bacteria are known to be the main constituent of the bog microbiome, co-occurring and interacting with archaea, fungi and other members of the microbiome [8, 12, 13]. Moreover, bog ecosystems were found to be natural hotspots for a high diversity of bacterial species and antagonists [12] and microbial genes [11] and interestingly also for antibiotic resistance genes [4, 11, 14]. A vast majority of bog bacteria were found to engage in a plant-associated, often endophytic life style [4]. The plant microbiota is involved in plant growth, productivity, adaptation, diversification and health [15–17], which is of special importance under extreme conditions in bog environments. Specifically, *Sphagnum* mosses were shown to contain a specific bacterial community which fulfils important roles for ecosystem functioning and health [1, 8, 12, 18–21]. A previous study indicated that *Acidobacteria*, *Actinobacteria* and *Proteobacteria* are highly responsive towards substrate-specific changes in the bog ecosystem [22]. Interestingly, studies indicate an extraordinary high degree of plant-specific microorganisms [11, 18, 23]; this can be explained by plant–microbe coevolution [17]. Coevolution was especially studied for microbial symbionts and pathogens, but the degree to which specialization and coevolution between plant species and microbes in ecosystems occur is relatively understudied [24]. For bogs, our hypothesis is that vascular plants with vascular tissues, which allow transport and diversification and which have more developed defence mechanisms against pathogens [25], harbour a more specific but less diverse microbiota than the phylogenetically older bryophytes.

Therefore, we studied bacterial community structures and functions in typical circumpolar bog vegetation of two Austrian bogs (Rotmoos and Pürgschachen Moor). We selected representative plants that belong to two separate phylogenetic branches, bryophytes, i.e. *Sphagnum magellanicum*, *Sphagnum angustifolium* and *Sphagnum fuscum* (peat mosses), *Pleurozium schreberi* (Brid.) Mitt. (Schreber’s big red stem moss), *Polytrichum strictum* (bog haircap moss) and *Mylia anomala* (anomalous flapwort; liverwort), and vascular plants, i.e. *Vaccinium myrtillus* (European blueberry), *Vaccinium oxycoccus* (bog cranberry), *Calluna vulgaris* (heather), *Andromeda polifolia* (bog rosemary), *Eriophorum vaginatum* (tussock cottongrass) and *Pinus mugo* (dwarf mountain pine), and analysed them using a shotgun metagenomic sequencing approach. In combination with targeted bioinformatics, such data can be used for functional microbiota profiling and to capture detailed taxonomic diversity of the entire microbiome [26]. Specifically, we addressed the following questions: (i) Are there general differences in bacterial communities between bryophytes and vascular plants? (ii) Do bacteria harbour functions that are specific for the different plant host interactions? and (iii) Do different bacterial taxa have distinct roles to support bog ecosystem functioning? Overall, this study provides key insights into the ecosystem function of bog-associated bacteria that are at present largely underrepresented in global surveys.

## Material and methods

### Sample collection and shotgun metagenomic sequencing

The samples for the analyses were obtained from northern Styria, Austria, in November 2012 within two geographically distinct peat bogs, Rotmoos (N47 41.029 E15 09.284 and N47 41.059 E1509.269) and Pürgschachen Moor (N47 34.835 E14 20.390 and N47 34.815 E14 20.482). In the past, the microbiome in these two bogs was intensively studied with a comprehensive experimental design [23, 27]. Based on these results, which showed markedly high species specificity of microbial communities, we developed a specific design for metagenomics approaches, which were already used to describe ecosystem functioning under extreme conditions as well as the role of archaea [11, 28]. At both sites, we selected randomly two plots (each of 1 m^2^) that represent the two typical plant communities dominated by either *Sphagnum magellanicum* or *Sphagnum angustifolium*. Bryophytes and vascular plants included in this study represent exemplary species that grow naturally in peat bogs; plant cover per plot together with environmental variables are detailed in Table S1. The metagenomic datasets (Table S2) were generated as previously described [11, 28]. In brief, total genomic DNA was extracted using the FastDNA Spin kit for soil (MP Biomedical, USA) following the manufacturer’s protocol. Shotgun metagenomic sequencing was performed by the sequencing provider Eurofins MWG Operon (Ebersberg, Germany). DNA library preparation was performed using a TruSeq DNA library kit (Illumina, USA) before the sequencing. The extracted DNA was then sequenced using an Illumina HiSeq 2500 system PE 2 × 150 bp (Illumina, USA) resulting in a range of 22.7–40.9 million reads per individual metagenome (Table S2). The datasets were previously used to explore archaeal community structure and function [28]. However, they remained entirely unexplored in terms of bacterial diversity which was previously one of the main constituents of bog microbiome [8, 27]. In the present study, the available raw data was subjected to a targeted bioinformatic approach to address this important component of the microbiome.

### Bacterial community structure and diversity analysis

Kraken2 v2.0.9 and Bracken v2.6.0 were used to measure the bacterial taxonomic composition and estimate the diversity of the short-read sequencing samples [29, 30]. Kraken2 implements a *k*-mer-based approach to classify individual metagenome reads by mapping all *k*-mer to the lowest common ancestor (LCA) of all genomes containing the given *k*-mer. The standard Kraken2 database was used to classify individual reads. Following Kraken2 taxonomic assignment, Bracken was used to estimate species abundances. To account for uneven sequencing depth, the datasets were normalized by rarefying to the lowest number of reads (2,271,071) and using MetagenomeSeq’s cumulative sum scaling (CSS; [31]) for subsequent alpha (species richness) and beta (variation in species composition) diversity analysis, respectively. Phyloseq, MicrobiomeAnalyst and vegan R packages implemented in RStudio v 1.3.1093 were used to analyse bacterial community diversity and composition [32–37]. Significant differences in alpha diversity estimates using the number of species (*S*) and Shannon index (*H*’) were determined by a non-parametric (ranked-based) Kruskal–Wallis test. The normalized Bray–Curtis dissimilarity matrix was subjected to analysis of similarities (ANOSIM) to test for significant effects of factors on microbial community structures. Biomarkers at bacterial order level between vascular plants and bryophytes were identified using a linear discriminant analysis effect size (LefSe) [38]. The threshold of Linear Discriminant Analysis (LDA) was set as 1 with cut-off *P*_adjusted_ values of 0.1.

### Non-redundant gene profiles of bog microbial communities

Default parameters were used for all mentioned software unless otherwise noted. The overall sequencing quality was assessed using FastQC v0.11.5 [39]. Based on the results, quality filtering was subsequently performed using Trimmomatic v0.39 and VSEARCH v2.14.2 to remove Illumina adaptor and low-quality reads (Phred < 20), respectively. The metagenome datasets were co-assembled using MEGAHIT v1.2.9 with meta-sensitive parameters [40]. Only contigs with a length > 1 kb were kept for subsequent analyses. Prodigal v2.6.3 was used to predict open reading frames of the assembled metagenome contigs [41]. Predicted protein-coding gene sequences were clustered into a non-redundant gene catalogue using CD-HIT-EST v4.8.1 at 95% nucleotide identity [42]. Representative gene sequences were then annotated using the blast algorithm in DIAMOND v0.9.35.136 through eggNOG-mapper v1.0.3 [43, 44] against the eggNOG database v5.0 [45] to reveal an ecologically relevant functional subsystem. Only reads mapped to contigs with KEGG Orthology (KO) annotation information were retained to calculate the abundances of genes and functional categories. To estimate gene abundance, the quality-filtered reads were mapped back to the abovementioned non-redundant gene catalogue using BWA v 0.7.17 and SamTools v1.7 [46, 47]. All non-bacterial contigs were removed before further analysis. In addition, low abundant genes (less than 100 reads) were removed to improve the sensitivity of differential gene abundance analyses. Differential gene abundance analysis was performed using edgeR as a suitable statistical method for the identification of differentially abundant genes in metagenomes [48, 49]. KO with log_2_ fold change > 1 and *P*_adjusted_ values below 0.1 were defined to be enriched or reduced between bryophytes and vascular plants. Beta diversity analysis (variation in KO composition) was performed as described for the taxonomic analyses. KO-pathway mapping was performed using the KEGG mapper [50].

### Detection of nitrogen cycling genes in metagenomic data

Reads from the metagenomes were aligned against NCycDB, a manually curated integrative database for nitrogen cycle gene profiling from shotgun metagenome sequencing data [51] using the blast algorithm in DIAMOND v0.9.35.136 [43]. We used a high confidence approach with a similarity threshold of 90% in combination with a stringent *E*-value < 10^−5^ to assign the metagenomic reads to nitrogen cycle genes [52]. Beta diversity and differential gene abundance analysis were performed as mentioned above.

### Reconstruction of bacterial metagenome-assembled genomes

Multiple binning methods, i.e. Maxbin2 v2.2.7, MetaBAT2 v2.12.1 and CONCOCT v1.1.0 [53–55], were used to reconstruct metagenome-assembled genomes (MAGs) as previously described [56]. The MAGs were then dereplicated using DASTool v1.1.1 [57]. CheckM v1.0.13 was used to calculate genome coverage, completeness and the percentage of contaminations in the MAGs [58]. Only medium-quality MAGs according to the current definition of the minimum information metagenome-assembled genome (MIMAG) standards [59] and with at least 80% completeness were kept for downstream analysis. Taxonomical classification of each MAG was identified using the Bin Annotation Tool (BAT) v4.6 [60]. Prediction of protein-coding genes and gene annotations were performed as described for the general metagenomic analyses. We further searched for virulence factors in the MAGs by implementing the Virulence Factors of Pathogenic Bacteria Database (VFDB; [61]). For the detection of potential virulence genes, a similarity threshold of 50% in combination with a stringent *E*-value < 10^−10^ was used to avoid false-positive hits [62]. We included clinical isolate genomes that are publicly available (Table S3) to compare virulence gene composition between the MAGs and them. Detailed phylogenetic analysis of the MAGs was performed using PhyloPhlAn v3.0 [63]. Abundance profiles of each MAGs were estimated by back mapping back the quality-filtered reads from each plant shotgun metagenomic dataset to MAG contigs using the same approach as described above. MAG abundance was quantified as mapped reads per kilobase per million reads (RPKM) divided by the metagenomic sample sizes (in millions of reads) and the length of the MAG in kilobases. Differences in MAG abundances between bryophytes and vascular plants were analysed using the pairwise Wilcox test. Counts of KO from each MAGs were used as input data to calculate distance matrix using Bray–Curtis dissimilarities. For analysing virulence gene compositions, a Jaccard dissimilarity matrix based on the presence/absence of these genes was used to generate hierarchical clustering [36]. The generated distance matrix was subjected for PERMANOVA to investigate if MAG phylogeny explains the composition of functional genes within the MAGs. The distance matrix was further visualized by using principal coordinate analysis (PCoA) plots. A heatmap plot was used to visualize the functional feature abundance profiles. Venn diagram was generated using InteractiVenn [64].

## Results

### Bacterial diversity with the bog vegetation

**Fig. 1:**
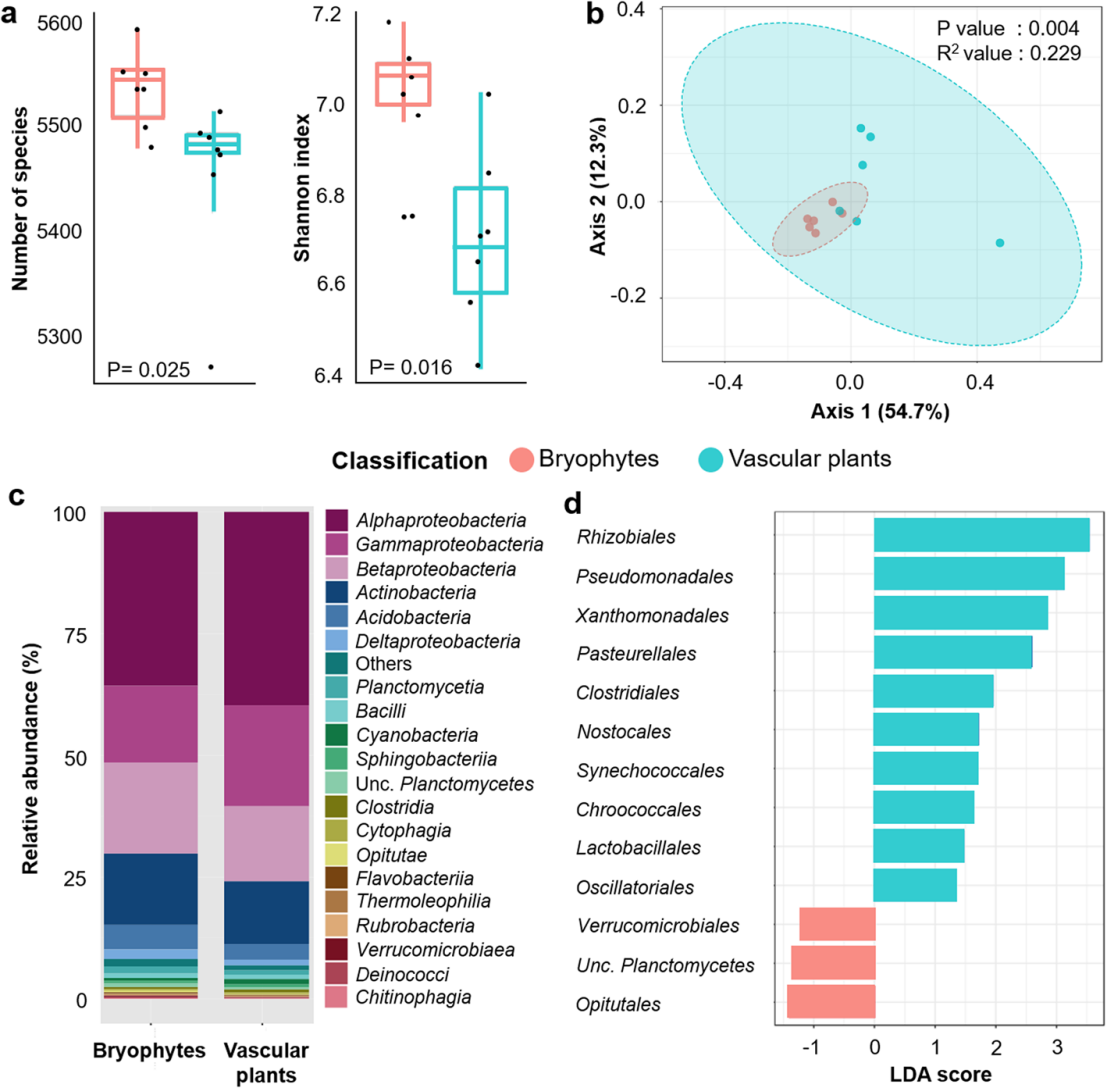
Bacterial richness, community clustering, composition and identified taxa that are either specific for bryophytes or vascular plants. Bacterial richness was assessed with the Shannon index (**a**). Community clustering of bacteria in bryophytes and vascular plants is visualized in a two-dimensional PCoA plot based on a Bray–Curtis matrix (**b**). Bacterial composition is shown at the class level (**c**). LEfSe analysis indicated specific biomarkers at bacterial order for both plant groups. Only bacterial taxa with LDA scores > 1 and *P*_adjusted_ < 0.1 according to LEfSe analysis are shown (**d**)

Targeted diversity analyses were conducted in order to resolve if bacterial community richness and structure differ between vascular plants and bryophytes although they grew in close proximity and all individuals were embedded into *Sphagnum* mosses. Alpha diversity analysis indicated that plant types (vascular plants and bryophytes) significantly affected bacterial richness and community structure (Fig. 1a). Based on the Kraken2 taxonomic classifier, a total of 5699 bacterial species was identified within the metagenome sequence library. Respective rarefaction curves indicate the sampling size was sufficient to capture overall bacterial diversity (Figure S1). A significantly higher number of species (*P* = 0.025) and Shannon index (*P* = 0.016) was observed in bryophytes (*S* = 5533, *H*’ = 7.0) in comparison to vascular plants (*S* = 5451, *H*’ = 6.7; Fig. 1a). A total of 37 core taxa with a relative abundance > 0.01% were exclusively detected in bryophytes. Beta diversity analysis revealed significantly different clustering between bacterial community structures of vascular plants and bryophytes (*R*^2^ = 0.229, *P* = 0.004; Fig. 1b). Additional beta diversity analyses were performed by analysing bacterial communities on phylum, order and genus levels (Figure S2). At higher taxonomic levels (phylum and class), the bacterial community composition was relatively similar between bryophytes and vascular plants (*R*^2^ = 0.175, *P* = 0.140; *R*^2^ = 0.130, *P* = 0.124; Figure S2a & b). However, a distinct clustering between the two groups was observed at lower taxonomic levels (order and genus) when subjected to the same analysis (*R*^2^ = 0.250, *P* = 0.015; *R*^2^ = 0.218, *P* = 0.004; Figure S2c & d).

Among bacterial phyla, *Proteobacteria* (74.1%) was the predominant taxonomic group in the bog vegetation followed by *Actinobacteria* (14.7%) and *Acidobacteria* (4.2%). Specific differences in bacterial composition were observed at lower taxonomic levels. A relatively higher proportion of *Alphaproteobacteria* and *Gammaproteobacteria* was observed for vascular plants (38.2 and 20.4%, respectively; Fig. 1c) in comparison to bryophytes (36.5 and 16.4%). In contrast, the bacterial class *Acidobacteria* was relatively more abundant in bryophytes (4.9%) compared to vascular plants (3.4%). A complementary LEfSe analysis indicated that several bacterial lineages within *Proteobacteria* (i.e. *Rhizobiales*, *Pseudomonadales*, *Xanthomonadales* and *Pasteurellales*) and *Cyanobacteria* (i.e. *Synechococcales*, *Nostocales*, *Synechococcales*, *Chroococcales*) were significantly enriched in vascular plants whereas bacterial lineages that belong to *Verrucomicrobia* (i.e. *Verrucomicrobiales* and *Opitutales*) and *Planctomycetes* (i.e. *Planctomycetales*) were significantly enriched in bryophytes (Fig. 1d).

### Profiling of bacterial functions in the bog microbiome

**Fig. 2:**
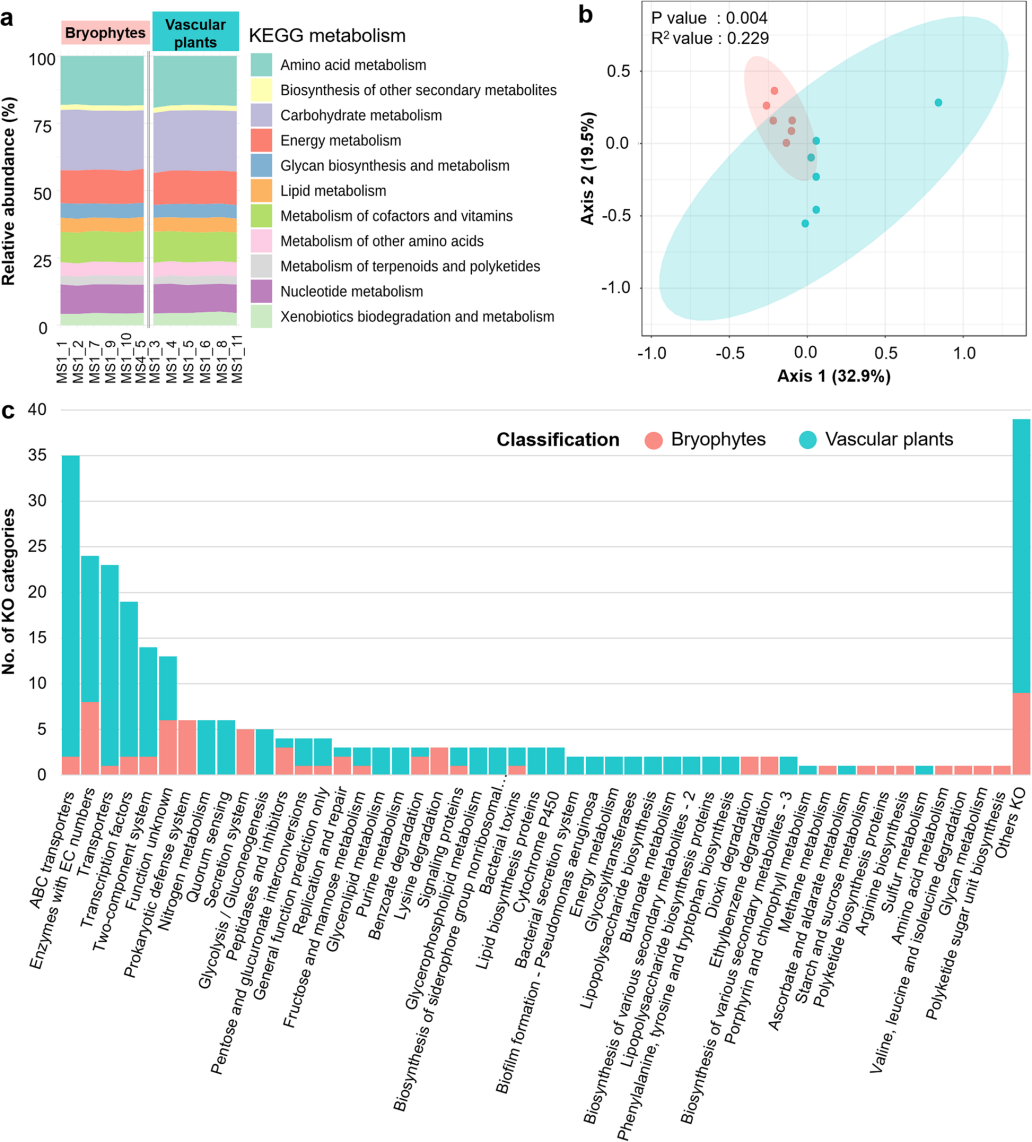
Comparison of functional assignment of the bog-associated microbiome, functional community clustering (**a**, **b**) and number of bacterial KEGG Orthology (KO) that enriched or depleted between bryophytes and vascular plants (**c**). Annotated genes were assigned to corresponding KEGG Orthology (KO) functional categories (**a**). A Bray–Curtis distance matrix between samples was visualized using a principal coordinate analysis (PCoA) plot (**b**). **c** Numbers of bacterial genes that were statistically enriched/depleted according to differential gene abundance analysis were obtained by using edgeR (log_2_ fold change > 1 and *P*_adjusted_ values below 0.1). These genes were grouped according to KEGG pathways (third tier) and visualized in a bar plot (**c**)

Due to the observed differences in the bacterial community structure, we expected that the overall functional profiles of the respective communities would be also different. Following filtering of metagenomic data, 8.56 × 10^6^– 27.9 × 10^6^ reads were annotated as bacterial genes (Table S2). Functional annotation assigned the reads into 5852 KOs. A high proportion of them (5522 KOs; 94.3%) was detected in all samples indicating a large functional core in the bog microbiome. The KOs were mainly associated with carbohydrate, amino acid and energy metabolism (22.1, 18.8 and 12.3%, respectively; Fig. 2a). Moreover, specific KOs were associated with functions known to be prevalent in plant-associated microbes, i.e. ABC transporters (*n* = 220, 3.8%), quorum sensing (*n* = 63, 1.1%), nitrogen metabolism (*n* = 41, 0.7%) and biosynthesis of siderophores (*n* = 20, 0.34%). Methane oxidizing bacteria are important to reduce methane emissions from peat bogs and provide CO_2_ for photosynthesis [1]. We also detected genes involved in methane oxidation, i.e. *pmoA*, *pmoB*, *pmoC* and *mmoX* that belong to *Alphaproteobacteria*. Altogether, beta diversity analysis indicated a clear clustering for microbiota’s functioning in bryophytes and vascular plants (*R*^2^ = 22.9%, *P* = 0.004; Fig. 2b).

A pairwise comparison using edgeR revealed that 290 KOs (log_2_ fold change > 1, *P*_adjusted_ < 0.1, Table S4) were significantly different between bryophytes and vascular plants. A total of 216 KOs was significantly enriched in vascular plants, whereas 74 KOs were reduced (Fig. 2c). Among the enriched KOs, 33 were assigned to ABC transporter pathways in vascular plants. Moreover, four KOs that were involved in oligosaccharide (i.e. galactose, raffinose and sorbitol), six KOs that were involved in monosaccharide (i.e. L-arabinose and xylitol) and six KOs that were involved in biotin and amino acid transporters (i.e. octopine and cystine) were enriched in vascular plants whereas two KOs assigned to bicarbonate transport system substrate-binding protein and cobalt/nickel transport system permease protein were enriched in bryophytes. A higher abundance of KOs (*n* = 6) that are related to the biosynthesis of other secondary metabolites (i.e. pinoresinol/lariciresinol reductase and antibiotic kanosamine) was also observed in vascular plants. In addition, a total of three KOs related to the biosynthesis of vitamins, i.e. vitamin K2, B1 and B12, were also enriched in vascular plants*.* The siderophore group represented by aerobacterin (K03897 and K03894), yersiniabactin (K04784) and mycobactin (K04789 and K04792) was also more abundant in vascular plants compared to bryophytes, whereas the siderophore enterobactin (k02361) showed the opposite trend. Within nitrogen metabolism, six KOs annotated as nitrogenase and nitrate reductase (*nifH*-K02588, *nifK*-K02591, *narG*-K00370, *narH*-K00371, *nifD*-KO2586 and *napA*-K02567) were enriched in vascular plants. Contigs that were assigned to *nifH* originated from *Alphaproteobacteria* and *Betaproteobacteria*. In contrast, KOs involved in degradation of lysine (*n* = 3), dioxin (*n* = 2) and aromatic compounds, i.e. aminobenzoate (*n* = 3), ethylbenzene (*n* = 2), naphthalene (*n* = 1), chlorocyclohexane (*n* = 1) and chlorobenzene (*n* = 1), were enriched in bryophytes. Notably, specific CRISPR-associated proteins (*n* = 6) were also enriched in the bryophytes indicating potentially higher selection pressures from bacteriophages in these plants (Fig. 2c). The overall results indicate that despite a high share of functional features, the plant hosts enrich not only certain taxonomic groups (Fig. 1d), but also distinct functional groups to support host functioning.

### Profiling of nitrogen cycling genes in the bog microbiome

**Fig. 3:**
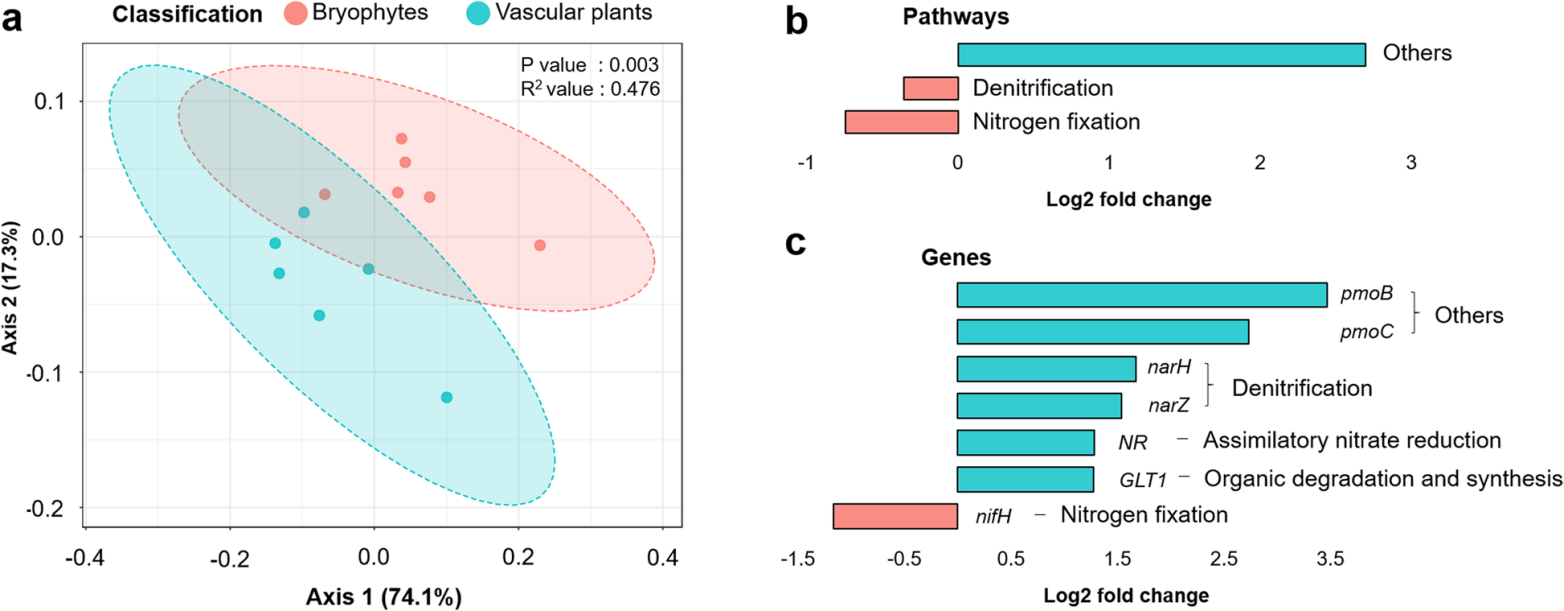
Clustering of nitrogen cycling gene profiles in vascular plants and bryophytes (**a**) and bar plots showing pathways (**b**) and genes (**c**) involved in nitrogen cycling that were enriched or reduced. A Bray–Curtis distance matrix between samples was visualized using a principal coordinate analysis (PCoA) plot (**a**). Pathways that were statistically enriched/reduced according to edgeR (*P*_adjusted_ < 0.1) were included (**b**). Nitrogen cycling genes that were statistically enriched/reduced according to edgeR (log_2_ fold change > 1 and *P*_adjusted_ < 0.1) were included (**c**). Negative log_2_ fold change values indicate that the respective genes are more abundant in bryophytes whereas positive values indicate that the genes are more abundant in vascular plants

As the nitrogen cycle is an important biogeochemical pathway in the bog ecosystem, we further explored if the respective communities (associated to bryophytes and vascular plants) were associated to distinct nitrogen cycling profiles. Using a high-stringency approach (90% similarity and < 10^−5^*E*-value), a total of 234,505 reads (ranging from 6417 to 36,098 reads per plant) was annotated as nitrogen cycling genes (Table S2). Beta diversity analysis indicated a distinct cluster between bryophytes and vascular plants (*R*^2^ = 47.6%, *P* = 0.003; Fig. 3a). Furthermore, a pairwise comparison using edgeR indicated that the abundance of genes that are involved in nitrogen fixation and denitrification was significantly higher in bryophytes compared to vascular plants (Fig. 3b, Table S5). However, the differences in log-fold change were only 0.7 and 0.4, respectively. When abundance profiles were analysed at the gene level, we observed that 11 genes that are involved in nitrogen cycling (Table S5) significantly differed between bryophytes and vascular plants. The abundance of the *nifH* gene was significantly higher in bryophytes. This result was in contrast to the abundance analysis using the eggNOG database (Table S4, see K02588-*nifH*) that indicated that the abundance of *nifH* was higher in vascular plants. A set of genes encoding particulate methane monooxygenase (*pmoB* and *pmoC*) which also have a shared high sequence similarity with the *amo* gene family [51] were enriched in vascular plants (Fig. 3). Genes encoding nitrate reductase (NAD(P)H) (*NR*), glutamate synthase [NADH] (*GLT1*) and nitrate reductase (*narH*) were also enriched in the vascular plants.

### Genome-centric analysis of prevalent bacteria in the bog vegetation

**Fig. 4:**
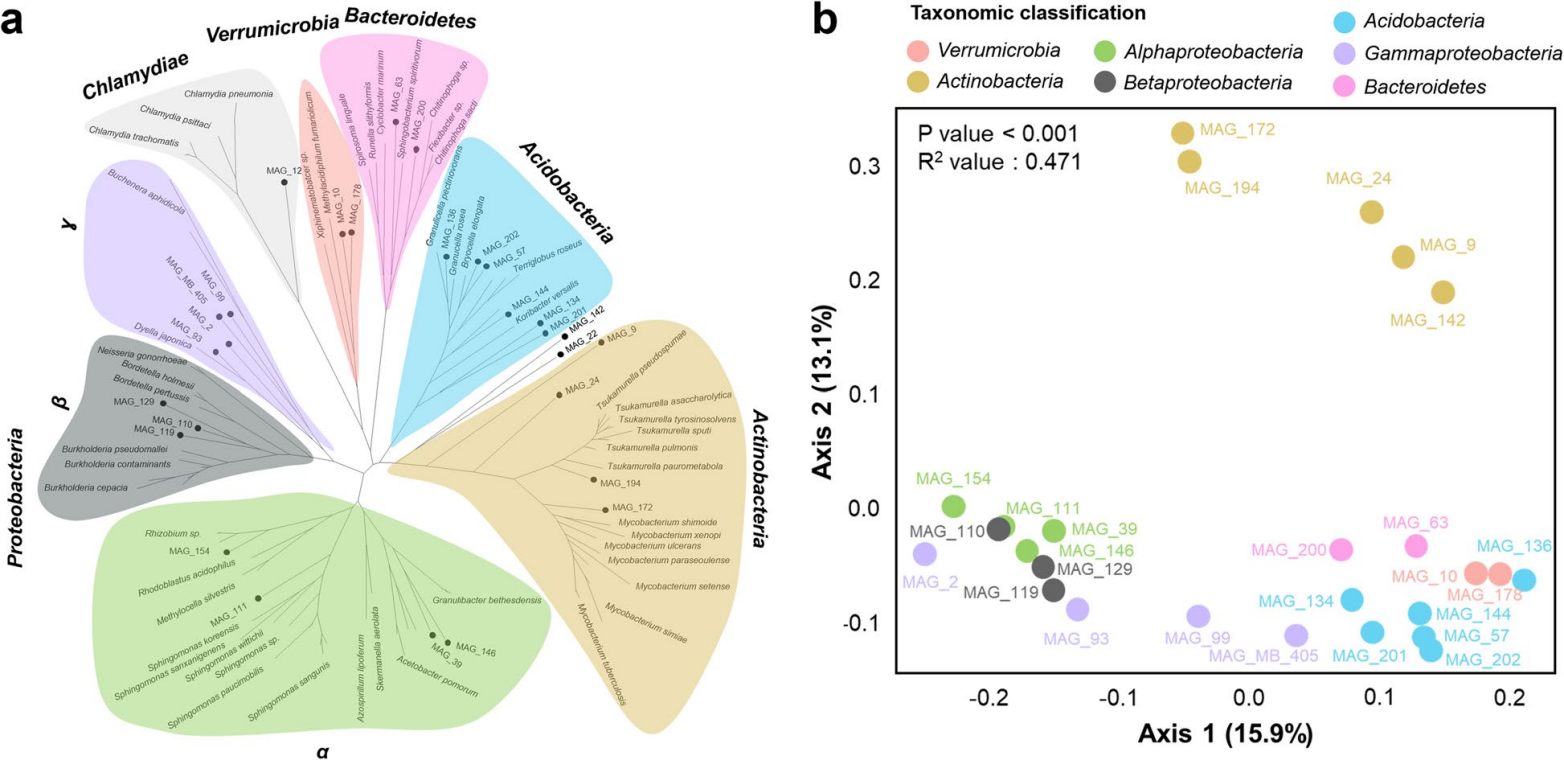
Phylogenetic tree of reconstructed metagenome-assembled genomes (MAGs) and clustering based on KEGG Orthology (KO) counts from each MAG. Different bacterial phylum and proteobacterial classes that are presented in the phylogenetic tree constructed MAGs are highlighted with different colours (**a**). Dots represent MAGs that were generated with metagenomic data from the bog ecosystem. Principal coordinate analysis (PCoA) of MAG clusters is based on KO counts in the genomes (**b**)

In total, 28 MAGs with completeness levels more than 80% and contamination levels lower than 10% were reconstructed from bog metagenome datasets representing the most abundant members of the bacterial communities. Taxonomical assignment (Fig. 4a, Table S6) MAGs (completeness > 80%) resulted in the classification to six phyla, i.e. *Proteobacteria* (*n* = 11), *Acidobacteria* (*n* = 6), *Actinobacteria* (*n* = 6), *Verrucomicrobia* (*n* = 2), *Bacteroidetes* (*n* = 2) and *Chlamydiae* (*n* = 1). One MAG was assigned to *Candidatus Eremiobacteraeota* which was previously known as Candidate division WPS-2 (bin ID: MAG_22). Despite high completeness (> 90%), several MAGs were only assignable at the class level, i.e. *Gammaproteobacteria* (MAG_2, MAG_99) and *Betaproteobacteria* (MAG_119, MAG_129, MAG_110), indicating a possible occurrence of novel bacterial lineages in the bog microbiome. Among MAGs, two MAGs (MAG_178 and MAG_201) that belong to *Verrucomicrobia* and *Acidobacteria*, respectively, were more abundant in non-vascular plants whereas four MAGs that belong to *Proteobacteria* (MAG_154, MAG_111, MAG_2 and MAG_119) were more abundant in vascular plants (*P*_adjusted_ < 0.1, Table S7). This result indicated that despite similar abundances from the majority of MAGs, the plant host seems to allow specific bacterial taxa to enrich as shown previously (Fig. 1d).

**Fig. 5:**
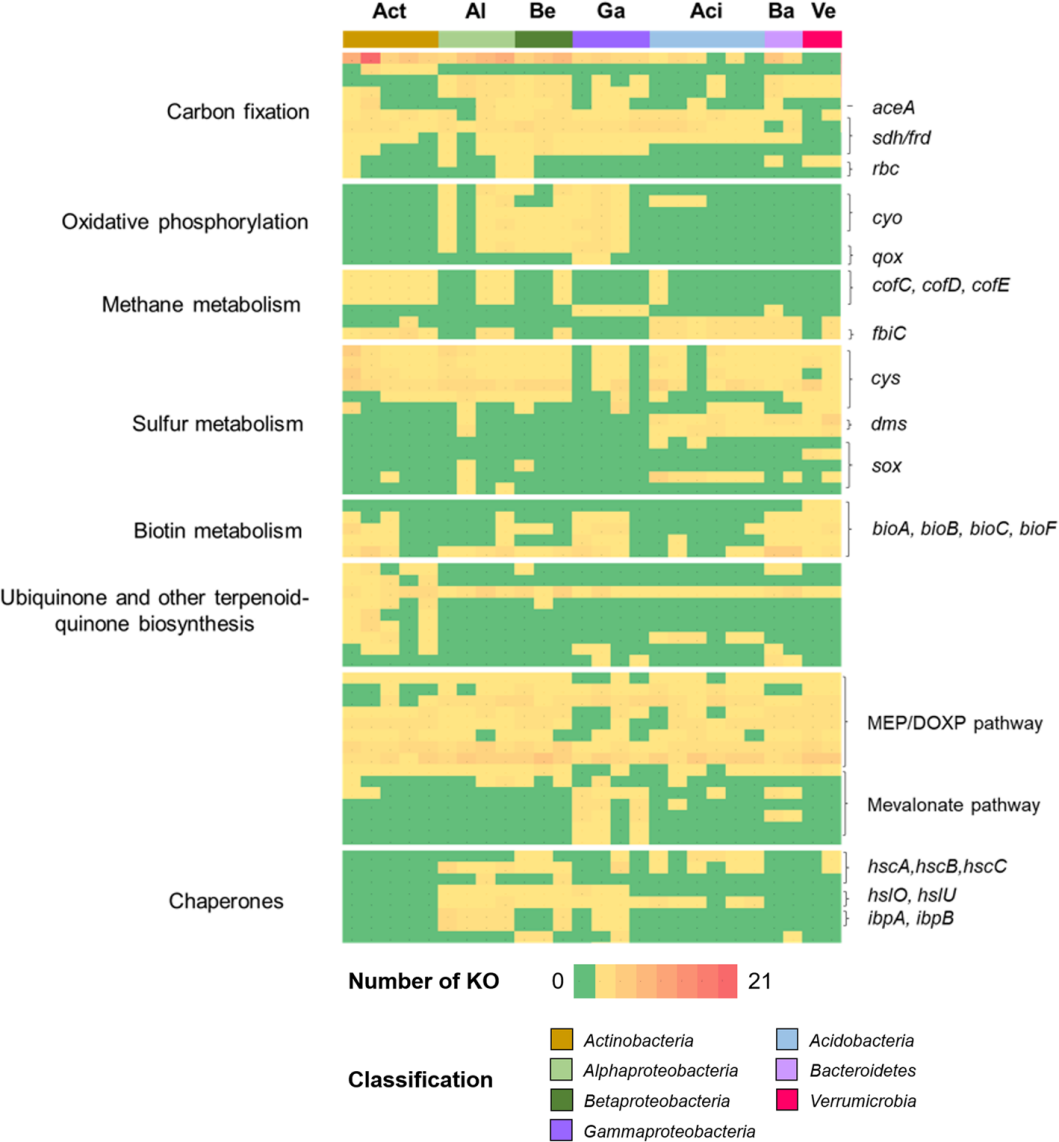
Heatmap plots showing abundance profiles for each bacterial group based on KEGG Orthology (KO) counts in MAGs. Different bacterial phyla and proteobacterial classes are highlighted with different colours. Act *Actinobacteria*, Al *Alphaproteobacteria*, Be *Betaproteobacteria*, Ga *Gammaproteobacteria*, Aci *Acidobacteria*, Ba *Bacteroidetes*, Ve *Verrucomicrobia*. The colour gradient from green to red represents a number of KO counts present in each MAG from low to high

A broad phylogenetic representation of bacteria and high genome completeness facilitated detailed assessments of the functional potential of different taxonomic groups in the bog vegetation. MAGs belonging to *Chlamydiae* (MAG_12) and *Candidatus Eremiobacteraeota* (Candidate division WPS-2*;* MAG_22) were excluded from the PERMANOVA analysis due to insufficient replicates for these groups (Fig. 4, Table S6). Function-based clustering in a PCoA plot indicated the occurrence of specific clusters was determined by MAG phylogeny (Fig. 3b). This was confirmed by a complementary statistical assessment with PERMANOVA (*P* < 0.001, *R*^2^ = 0.471). Three distinct clusters were observed, i.e. cluster 1 (*Actinobacteria* MAGs), cluster 2 (*Alpha*-, *Beta*- and *Gammaproteobacteria*) and cluster 3 (*Acidobacteria*, *Bacteroidetes* and *Verrucomicrobia*). This observation indicates that various functional groups are present within naturally occurring bacterial communities in the bog vegetation. In a subsequent step, functions that differed between MAGs (Fig. 5) and that might be involved in plant–microbe interactions were explored in detail. *Alpha*-, *Beta-*, *Gammaproteobacteria* and *Actinobacteria* encoded a higher number of genes involved in carbon fixation pathways, i.e. *atoB* (acetyl-CoA C-acetyltransferase) and *sdhD.* In fact, the majority of MAGs that belong to *Alpha*-, *Beta-*, *Gammaproteobacteria* and *Actinobacteria* had a complete gene set of *sdhABCD*/*fdrABCD*. The latter taxa also had a higher number of genes involved in carbon fixation pathways, i.e. *mcl* (citramalyl-CoA lyase), than other MAG groups whereas *Alpha*-, *Beta-* and *Gammaproteobacteria* harboured a higher number of *pta* gene encode phosphate acetyltransferase. Moreover, we also detected small and large subunits of RuBisCO genes and *aceA* which encodes isocitrate lyase in three MAGs (MAG_39, MAG_129 and MAG_194) that were assigned to *Alpha*-, *Betaproteobacteria* and *Actinobacteria*. A set of genes that are involved in oxidative phosphorylation, especially cytochrome o ubiquinol oxidase (*cyo*) and cytochrome aa3 quinol oxidase (*qox*), were found to be more prevalent in MAGs of the *Proteobacteria* phylum compared to others (Fig. 5). *Proteobacteria* and *Actinobacteria* might play a crucial role in carbon fixation in the bog ecosystem (Fig. 5). The latter taxa also had a set of genes that are involved in F420 biosynthesis, which plays a central role in methanogenesis due to the presence of *cofC*, *cofD*, *cofE* and *fbiC*. Genes involved in assimilatory sulfate reduction were evenly distributed (*cysN*, *cysD*, *cysNC*, *cysI* and *cysJ*) among MAGs. However, *Acidobacteria* and *Verrucomicrobia* harboured a higher number of genes related to sulfur metabolism through the Sox system such as *soxB* and *soxX*, respectively (Fig. 5). Genes that are involved in assimilatory/dissimilatory nitrate reduction such as *narB*, *nirA*, *nirB* and *nirD* were detected in MAGs that belong to *Actinobacteria* (MAG_194 and MAG_172), *Alphaproteobacteria* (MAG_111), *Betaproteobacteria* (MAG_129 and MAG_110) and *Gammaproteobacteria* (MAG_2).

In addition to genes from the central carbohydrate and energy metabolism, also, genes that are related to vitamin biosynthesis, metabolism of secondary metabolites and abiotic stress responses and thus potentially important for host-microbe interactions were detected. A set of genes (*bioA*, *bioB*, *bioC* and *bioF*) that is involved in biotin metabolism was detected in MAGs that belong to *Actinobacteria*, *Gammaproteobacteria*, *Bacteroidetes* and *Verrucomicrobia*. Specific genes involved in menaquinone biosynthesis (*menA*, *menB*, *menC*, *mend* and *menF*) were prevalent in *Actinobacteria*. A collection of genes involved in the MEP/DOXP pathway (*dxr*, *ispA*, *ispB*, *ispD*, *ispE*, *ispF*, *gcpE*, *ispH* and *idsA*) were detected in the majority of MAGs, except from those that belong to *Gammaproteobacteria*. Interestingly, *Gammaproteobacteria* may also play an important role in the production of terpenoid but through the mevalonate pathway due to the presence of *idi*, *mvaA*, *mvaD*, *mvk* and *mvkA2* genes (Fig. 5). Several molecular chaperones, i.e. *hslO*, *ibpA*, *ibpB* and *pccA*, were more prevalent in MAGs that belong to *Proteobacteria*, which indicates their enhanced ability to mitigate environmental stress.

Among the MAGs, two MAGs (MAG_178 and MAG_201) that were assigned to *Verrucomicrobia* and *Acidobacteria*, respectively, were more abundant in bryophytes. In contrast, four MAGs that belong to *Proteobacteria* (MAG_154, MAG_111, MAG_2 and MAG_119) were more abundant in vascular plants (*P*_adjusted_ < 0.1; Table S7). A key feature that was found in MAG_178 and MAG_201. was that these MAGs harboured a complete set of enzymes that are involved in riboflavin (B2) biosynthesis, i.e. GTP cyclohydrolase II and riboflavin synthase. On the other hand, all of the MAGs that were more prevalent in vascular plants harboured genes that encode urea transport system permease protein (*urtA*, *urtB*, *urtC*, *urtD* and *urtE*). In particular, MAG_154 also harboured genes that encode multiple sugar transport system ATP-binding proteins. These results indicate that despite similar abundances of the majority of analysed MAGs, that plant host likely facilitates colonization of bacterial taxa with specific functions (Fig. 1) to support plant host physiology.

Previous studies provided first indications that the keystone species in the bog ecosystem, *Sphagnum*, is a reservoir for potentially opportunistic human pathogens [14, 65]. In this study, we also identified a list of potential human pathogens, i.e. *Clamydia* and *Mycobacterium spec. div*. Therefore, we searched for potential virulence factor homologs (≥ 50% identity and < 1e − 10 *E*-value) that are prevalent among the MAGs (Table S8). Calculation of the average distance of the MAGs and clinical isolate genomes indicated cluster formation that was mainly attributable to bacterial class level (Figure S3). This result was strengthened by PERMANOVA analysis which indicated that the origin of isolates (clinical and environment) only explained a small variation virulence gene composition (*R*^2^ = 2.4%) while taxonomical identity explained 29.6% of the variation. There was no clear differentiation in the virulence gene composition between the genomes of closely related clinical and environmental isolates within the clusters. Nevertheless, we identified a number of genes that were exclusively detected in the bog MAGs in comparison to the closely related clinical isolate genomes. For instance, when MAG 172 was compared with a clinical *Mycobacterium*, MAG12 with clinical *Chlamydia* isolates and MAG_111 with clinical *Sphingomonas* isolates, bog-specific differences were found (Figure S4; Table S9). When analysed in detail, a gene that encodes catalase peroxidase (*katA*) was detected in MAGs 110 and 172 but was not present in the clinical isolates. Specific genes that are involved in iron uptake, i.e. *ccm*, *ybtQ* and *mbtJ*, were also only present in MAGs generated from the bog ecosystem when compared to their closely related clinical taxa. Genes encoding the phytotoxin phaseolotoxin were exclusively detected in MAGs 111 and 139 that belong to *Sphingomonadaceae* and *Betaproteobacteria*, respectively.

## Discussion

**Fig. 6:**
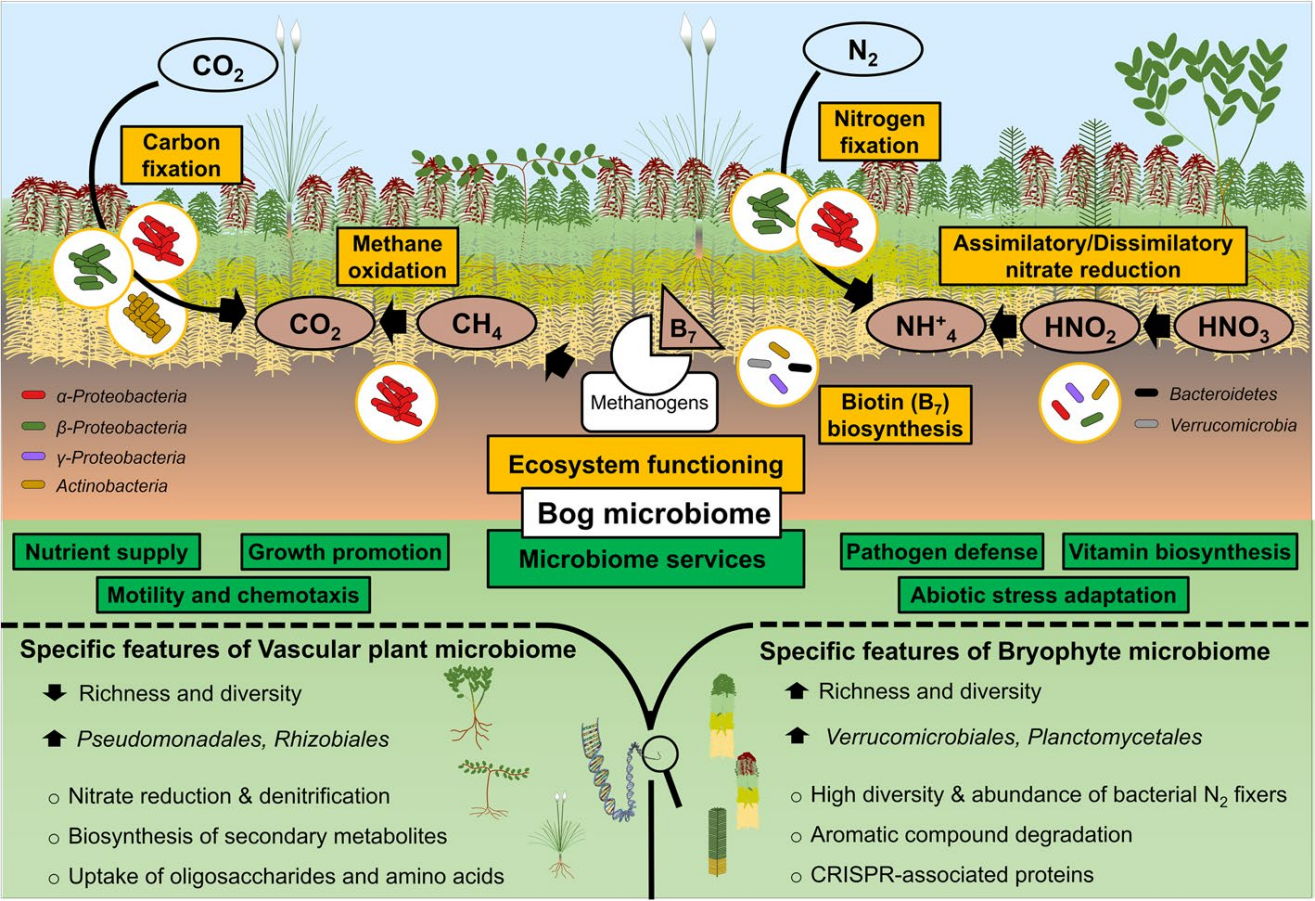
Schematic illustration of bacterial groups associated with vascular plants and bryophytes contributing to bog ecosystem functioning. Different bacterial groups involved in bog ecosystem functioning are indicated by different colours (red: *Alphaproteobacteria*, green: *Betaproteobacteria*, purple: *Gammaproteobacteria*, gold: *Actinobacteria*, blue: *Acidobacteria*, black: *Bacteroidetes* and grey: *Verrucomicrobia*). Overall microbiome services and plant-specific (for bryophytes and vascular plants) traits were included in the lower part of the figure

In the present study, we showed that vascular plants and bryophytes enrich certain bacterial taxa with distinct functional properties reflecting plant–microbe coevolution. The differences in community composition were statistically significant despite the fact that all analysed plant species co-occur in close proximity and are connected by a bacterial meta-community [11]. Plant–microbe coevolution was already reported for distinct plants, interactions and processes [17, 24]; here, we show complementary implications at the ecosystem level by employing a shotgun metagenomic sequencing approach using a novel bioinformatic pipeline and detailed genome-centric analysis. Compared with previous studies, we found concordance in terms of the prevalence of *Proteobacteria*, but also observed differences in the relative abundance of less abundant taxa [11]. This is commonly observed when studies based on the amplification of marker genes are compared with such based on shotgun metagenomics [66–68]. In this context, it is noteworthy to mention that using shotgun metagenomics we detected a higher number of bacterial taxa (5699 vs. 4898 bacterial OTUs/species). Bogs are important native models to study general rules of plant–microbe coevolution and specificity, because these ecosystems have been stable for centuries and are shaped by extreme but similar abiotic factors around the world [69–71]. In addition to confirming previous findings with complementary approaches, we further illustrated that the bog microbiome is collectively involved in beneficial functions, which are referred to as microbiome services, i.e. nutrient supply, plant growth promotion and vitamin biosynthesis to support bog ecosystem functioning (Fig. 6).

Bryophytes were found to be associated with a significantly higher bacterial richness and diversity in comparison to vascular plants. Nevertheless, the bacterial alpha diversity was found to be in a similar range for both plant systems. We argue that the spatial proximity of plants facilitates the transfer of microorganisms, leading to an increase in overall diversity while maintaining plant group specificity. Bryophytes, including *Sphagnum* mosses, provide an exceptional habitat for plant-associated bacteria and their richness is tightly connected with the bog ecosystem functioning [27]. A recent study showed that a decline in the microbial diversity of *Sphagnum* mosses was correlated with a decrease in N_2_ fixation activity [72], which highlights the importance of bacterial richness for the overall bog ecosystem functioning. The community composition was more similar among different bryophytes, and more different between the vascular plant species. The latter are known to recruit a specific fraction of bacterial taxa from their surrounding environment due to secretory activities in their root system [16], which is also suggested as an entry point for bacteria to colonize above-ground plant compartments [73]. In vascular plants, bacterial diversity is commonly reduced from soil to rhizosphere to endosphere [16]. In contrast, bryophytes, which lack functional root structures, do not rely on their belowground compartments to recruit bacteria. Processes of water and mass transfer run over the entire surface of the plants. They can maintain a high bacterial diversity within the whole lifecycle, independent of their respective environment [23]. A foregoing study indicated that the liverwort *Marchantia paleacea* harboured a higher bacterial diversity than its soil substrate [74]. Vascular plants are more influenced by host-environmental interactions especially in the bog ecosystem with its extreme conditions, i.e. low pH, nutrient and high water saturation [4, 23].

When bacterial community composition was explored in the present study, a subset of differing bacterial features was detected between vascular plants and bryophytes indicating that the analysed plant hosts harboured functionally adapted bacterial communities. In the bog ecosystem, vascular plants and bryophytes grow in close proximity and are mostly embedded in the predominant *Sphagnum* plants. Therefore, it was to be expected that a majority of the prevalent bacteria and their functions, i.e. ABC transporters, quorum sensing, nitrogen metabolism and biosynthesis of siderophores, are widely shared by hosts as already shown before by marker gene analyses [8, 12, 28]. Nevertheless, plant-specific differences in the bacterial community, especially at the bacterial order level and below, as well as their function were observed. Previous studies have provided concordant results for specific functional groups in these environments [23, 75, 76]; however, they did not assess the entire taxonomic and functional diversity in typical bog ecosystems. Coevolution between microbes and hosts has apparently led to nuanced functional specificity in different plant groups. We detected a higher abundance of bacterial genes involved in aromatic compound degradation in bryophytes that might be explained by the typical accumulation of the aromatic compound in *Sphagnum* bogs that can be used as a carbon source by bacteria [77, 78]. In nutrient-poor environments, bryophytes such as *Sphagnum* mosses can absorb and accumulate mineral nutrients all over their surface highly effective even in extreme nutrient-poor environments, which gives them a competitive advantage over the vascular plant [79]. In contrast, root systems of typical vascular plants in bog ecosystems, e.g. *Eriophorum vaginatum*, *Calluna vulgaris*, *Vaccinium myrtillus* and *Vaccinium oxycoccus* that were included in the present study, can penetrate into deeper portions of the bog with their root systems [6, 28]. ABC transporter genes that are involved in the uptake of oligosaccharides and amino acids were enriched in the vascular plants. Enrichment of bacteria with this function might be more important for microbes of vascular plants growing in bogs to increase nutrient uptake from the environment and support their growth under nutrient-limited conditions.

Bacterial communities of bryophytes and vascular plants were shown to harbour different nitrogen cycling gene profiles. In nitrogen-poor environments, bacteria play a crucial role in nitrogen acquisition for the growth and development of plants (reviewed in [80, 81]). Nitrogen-fixing bacteria with *nifH* genes were shown to be more prevalent in bryophytes in comparison to vascular plants when an extended reference database was used. It should be noted that when solely using the eggNOG database, the abundance of *nifH* genes was found to be higher in vascular plants. However, the read number that was identified by using the eggNOG database was substantially lower (898 reads) when compared to NCycDB database hits (7608 reads). NCycDB was constructed by integrating nitrogen cycling genes from various databases (COG, KEGG, SEED and eggNOG) and thus provides a better reference set for assignments. This result also highlighted that bryophytes, especially *Sphagnum*, contained a high diversity of bacterial genes involved in nitrogen fixation. It is in agreement with previous findings describing the potential of nitrogen-fixing bacteria in bogs and possible occurrence of novel *nifH* variants [27]. Hence, this observation corroborates the importance of nitrogen-fixing bacteria which were mainly assigned to *Alphaproteobacteria*, for *Sphagnum* mosses [4, 8, 12]. In contrast to the observed abundance of *nifH* genes, a higher number of genes encoding nitrate reductases for denitrification processes were observed in metagenomes of vascular plants. The results are in accordance with previous findings that different plant species harbour specific microbial communities with specific functions even when grown on the same environment [82, 83].

By including a detailed genome-centric analysis of microbial communities, we were able to identify the metabolic potential of individual MAGs and highlight the specific roles of each functional guild. *Alpha*-, *Beta*- and *Actinobacteria* likely play an important role in carbon fixation in this ecosystem. Previous studies have shown that autotrophic bacteria within the bacterial phyla *Proteobacteria* and *Actinobacteria* significantly contribute to carbon fixation in various environments [84, 85]. Furthermore, they account for up to 4% of the total CO_2_ fixed by terrestrial ecosystems each year [86]. To our knowledge, so far, only few studies reported this bacterial feature from the bog ecosystem [87, 88]. Here, we showed that novel lineages of *Betaproteobacteria* and *Actinobacteria* that are found in the bog also harbour similar features involved in carbon fixation. MAGs that were assigned to these taxa are likely capable to fix carbon via RuBisCo. They were also shown to possess isocitrate lyase, a key enzyme involved in the glyoxylate shunt [88]. In analogy to our study, this strategy to fix carbon, which likely occurs when carbohydrates from the plant that are needed for biomass production by the bacteria are scarce, has been previously described in a novel bacterial lineage associated with moss species and named candidate phylum *WPS-2* [87]. *Alpha*-, *Beta*-, *Gammaproteobacteria* and *Actinobacteria* likely play important roles in controlling and balancing the amount of available nitrogen in the bog ecosystem due to the presence of genes that are involved in assimilatory/dissimilatory nitrate reduction. *Sphagnum* can efficiently uptake ammonium and use it as the main nitrogen source [89, 90]; thus, *Alpha*-, *Beta*-, *Gammaproteobacteria* and *Actinobacteria* likely provide additional available ammonium through assimilatory/dissimilatory nitrate reduction. In addition, our data suggests that *Acidobacteria*, *Bacteroidetes* and *Verrucomicrobia* play an important role in sulfur metabolism by catalysing the oxidation of thiosulfate to sulfate employing the Sox system [91], which was indicated by the presence of *soxB*, *soxX* and *soxY* genes. Overall, we could detect specific functional guilds that fulfil distinct roles related to key nutrient cycles in the bog ecosystem.

Other detected functions such as vitamin supply and resistance against biotic and abiotic stress factors indicate interactions with the host plants. *Actinobacteria*, *Gammaproteobacteria*, *Bacteroidetes* and *Verrucomicrobia* were shown to take part in biotin metabolism that might be also needed for stimulating the growth of some methanogens [92, 93]. Our findings suggest that *Actinobacteria* are involved in vitamin K2 biosynthesis due to the presence of genes involved in menaquinone biosynthesis. Vitamin K2 is involved in photosynthesis as a predominant electron carrier under low oxygen concentration [94, 95], a typical condition at the surface of peatlands [4]. Terpenoid synthesis genes that were identified within MAGs indicated clear segregation into mevalonate and MEP/DOXP (non-mevalonate) pathways. Moreover, *Proteobacteria* were shown to be well equipped with defence mechanisms against oxidative stress and heat shock due to a frequent occurrence of genes encoding molecular chaperones Hsp33 (*hslO*) and Hsp20 (*ibpAB*) [96, 97].

Interestingly, we also detected diverse putative virulence genes with high homology to sequences in the MAGs. The identified virulence genes allow human pathogenic bacteria to establish infections, survive in the hostile host environment and cause diseases [61]. However, in nature, the virulence genes such as flagella, the O-antigen of lipopolysaccharides and molecular chaperones facilitate bacterial colonization of their host plants [98, 99]. Previously, potential facultative pathogens were isolated from bog environments [14, 65]. This study reinforces previous observations that there is no clear separation between clinical and environmental isolates [100]. A similar result was previously reported in a study by Lira and colleagues [101]. When comparing clinical and environmental genomes of *Stenotrophomonas maltophilia* strains, they found that genomic composition is not sufficient to differentiate clinical and environmental isolates. Therefore, the presence of virulence genes that encode adherence-related proteins, stress-related proteins and iron uptake proteins may be used by the MAGs to overcome the plant defence system and efficiently colonize the host plants, especially vascular plants, in their natural environment [102]. However, these genes can become pathogenicity factors when transferred to other environments. In the present study, we could even show that some environmental isolates can harbour more virulence factors than their clinical counterparts, which further confirms that many human pathogens likely originated in the environment.

## Conclusion

Differences in the abundance of specific bacterial taxa with distinct functions between vascular plants and bryophytes indicate coevolution with their host plants. By employing genome-centric analyses, we could identify a highly complex metabolic potential within the bog microbiome and expand our understanding of the role of microorganisms in bog vegetation. The extraordinary high functional microbial diversity discovered in bog ecosystems allows understanding of stability, functioning and ecosystem health in bogs. However, this includes a high diversity of antimicrobial resistance as well as virulence genes, which indicates not only a reservoir for multi-resistant pathogens but also a source for studying suppression of them under natural conditions and emergence under anthropogenic influence.

## Supplementary Information

**Additional file 1: Figure S1.** Rarefaction curves showing the number of metagenomic reads that were classified as bacterial sequences according to the Kraken2 classifier. **Figure S2.** Bacterial community clustering at phylum (a), class (b), order (c) and genus (d) level was visualized in two-dimensional Bray Curtis PCoA plots. **Figure S3.** Analysis of virulence gene composition of clinical and environmental bog isolates. Hierarchical clustering of clinical and environmental strains is based in the presence/absence of virulence genes in the genome. **Figure S4.** Venn diagrams showing the numbers of shared and unique virulence genes detected in clinical and environmental bog isolate genomes. The graph was generated using InteractiVenn [61]. **Table S1.** Description of vegetation field plots regarding habitat characteristics and plant coverage per plot. **Table S2.** List of the 12 plant species that were included to represent the vegetation of a *Sphagnum*-dominated bog ecosystem. **Table S3.** Detailed taxonomical classification, source of origin and NCBI accessions of clinical and environmental isolate genomes. **Table S4.** List of the KEGG orthologs that were significantly enriched between bryophytes and vascular plants. **Table S5.** List of the pathways and genes involved in nitrogen cycling that were significantly enriched in either bryophytes or vascular plants. **Table S6.** Detailed taxonomic classification, completeness, contamination values, and genome sizes of bacterial MAGs. **Table S7.** Abundance estimation of metagenome assembled genomes in each plant sample. **Table S8.** Prevalence of putative virulence factors as predicted for metagenome-assembled genomes (MAGs) using the virulence factor database (VFDB). **Table S9.** Putative virulence factors that were uniquely present in the MAGs generated from the bog ecosystem when compared to their closely related clinical isolates.

## Data Availability

This shotgun metagenome project has been deposited in the European Nucleotide Archive (ENA) database under the study number PRJEB39100 and accession numbers ERR4298333 and ERR4298344.
