# Supplementary material for: Bog ecosystems as a playground for plant–microbe coevolution: bryophytes and vascular plants harbour functionally adapted bacteria
Source: Microbiome. 2021 Aug 11;9:170. doi: 10.1186/s40168-021-01117-7 (PMC8359052; doi:10.1186/s40168-021-01117-7)
Supplement: Supplementary file 2 — Additional file 1: Figure S1. Rarefaction curves showing the number of metagenomic reads that were classified as bacterial sequences according to the Kraken2 classifier. Figure S2. Bacterial community clustering at phylum (a), class (b), order (c) and genus (d) level was visualized in two-dimensional Bray Curtis PCoA plots. Figure S3. Analysis of virulence gene composition of clinical and environmental bog isolates. Hierarchical clustering of clinical and environmental strains is based in the presence/absence of virulence genes in the genome. Figure S4. Venn diagrams showing the numbers of shared and unique virulence genes detected in clinical and environmental bog isolate genomes. The graph was generated using InteractiVenn [61]. Table S1. Description of vegetation field plots regarding habitat characteristics and plant coverage per plot. Table S2. List of the 12 plant species that were included to represent the vegetation of a Sphagnum-dominated bog ecosystem. Table S3. Detailed taxonomical classification, source of origin and NCBI accessions of clinical and environmental isolate genomes. Table S4. List of the KEGG orthologs that were significantly enriched between bryophytes and vascular plants. Table S5. List of the pathways and genes involved in nitrogen cycling that were significantly enriched in either bryophytes or vascular plants. Table S6. Detailed taxonomic classification, completeness, contamination values, and genome sizes of bacterial MAGs. Table S7. Abundance estimation of metagenome assembled genomes in each plant sample. Table S8. Prevalence of putative virulence factors as predicted for metagenome-assembled genomes (MAGs) using the virulence factor database (VFDB). Table S9. Putative virulence factors that were uniquely present in the MAGs generated from the bog ecosystem when compared to their closely related clinical isolates. [file 40168_2021_1117_MOESM2_ESM.docx]

**Supplementary Material**

**Bog ecosystems as a playground for plant-microbe coevolution: bryophytes and vascular plants harbour functionally adapted bacteria**

**Wisnu Adi Wicaksono^a^, Tomislav Cernava^a#^, Christian Berg^b^, Gabriele Berg^a#^**

*^a^Institute of Environmental Biotechnology, Graz University of Technology, Graz, Austria*

^b^*Institute of Plant Sciences, University of Graz, Graz, Austria*

^#^Corresponding authors:

Tomislav Cernava & Gabriele Berg, Graz University of Technology, Graz

Emails: [tomislav.cernava@tugraz.at](mailto:tomislav.cernava@tugraz.at) & gabriele.berg@tugraz.at

**Authors’ email addresses:** wisnu.wicaksono@tugraz.at, christian.berg@uni-graz.at

**Short title:** Microbiome of bog plants

**Submitted to:** Microbiome

**Keywords:** bog ecosystems, bryophytes, vascular plants, microbiome, coevolution

**Supplementary Figures**

**
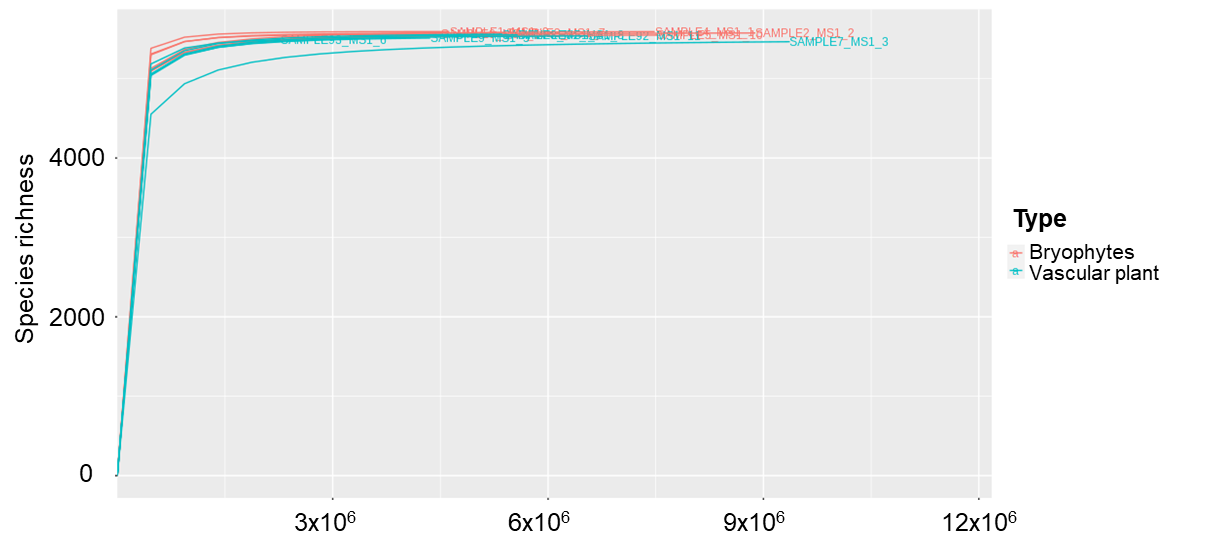
**

**Fig. S1.** Rarefaction curves showing the number of metagenomic reads that were classified as bacterial sequences according to the Kraken2 classifier.


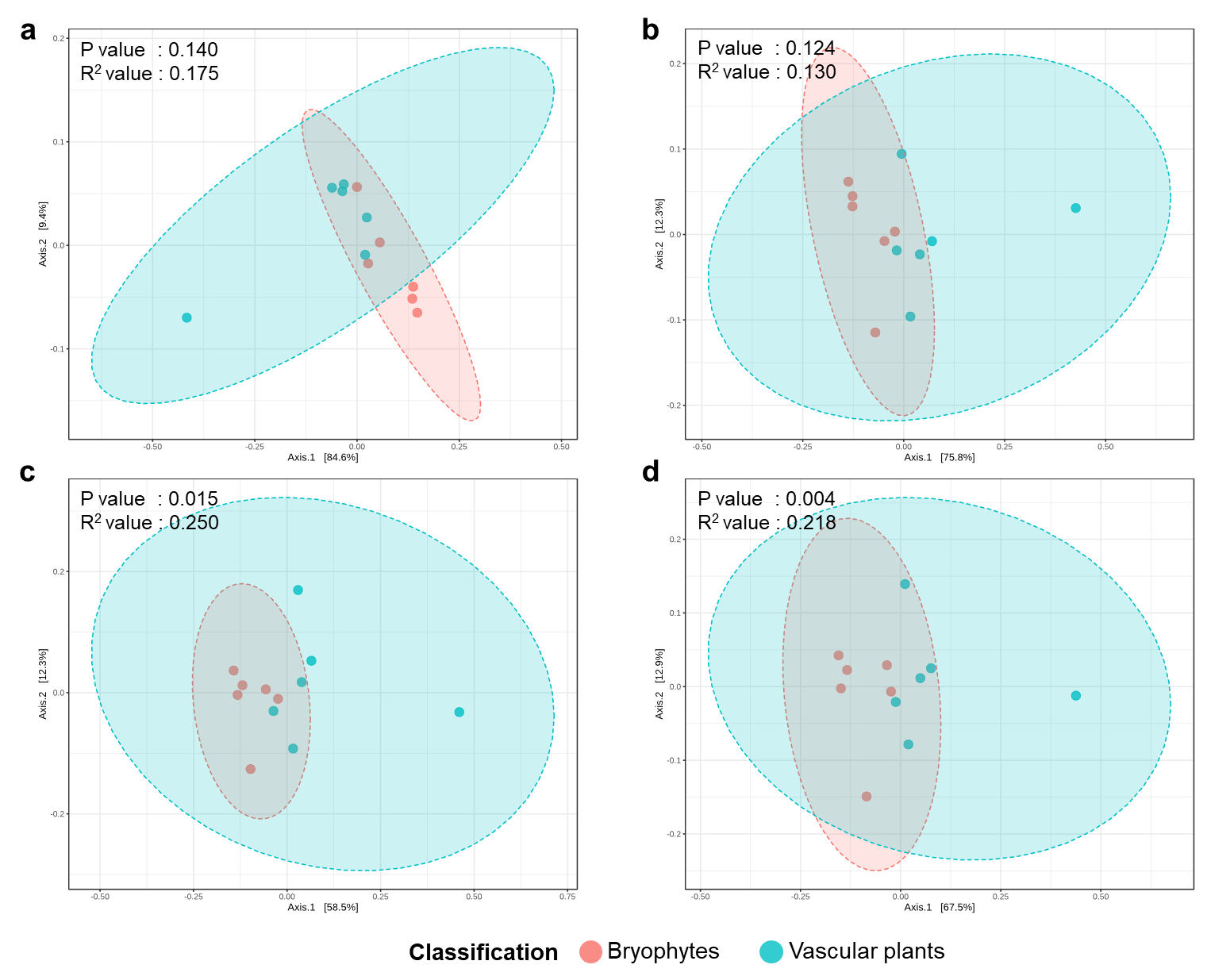


**Fig. S2.** Bacterial community clustering at phylum (a), class (b), order (c) and genus (d) level was visualized in two-dimensional Bray Curtis PCoA plots.

**
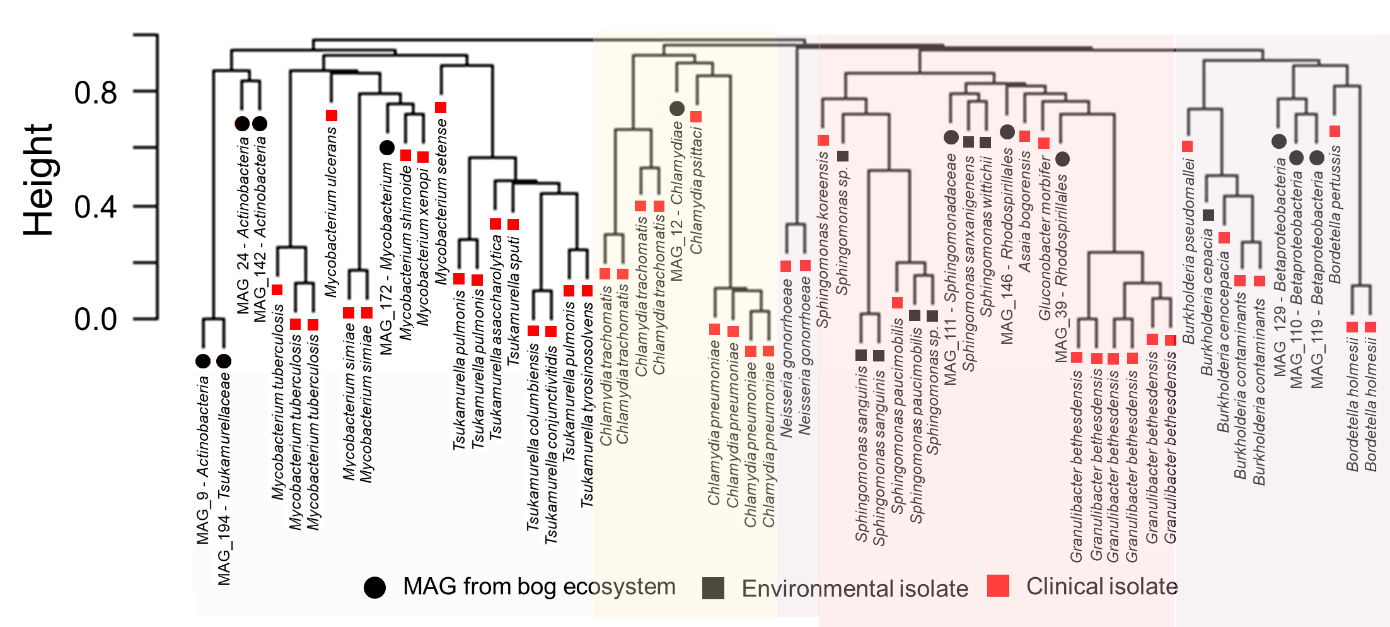
**

**Fig. S3.** Analysis of virulence gene composition of clinical and environmental bog isolates. Hierarchical clustering of clinical and environmental strains is based in the presence/absence of virulence genes in the genome.

**
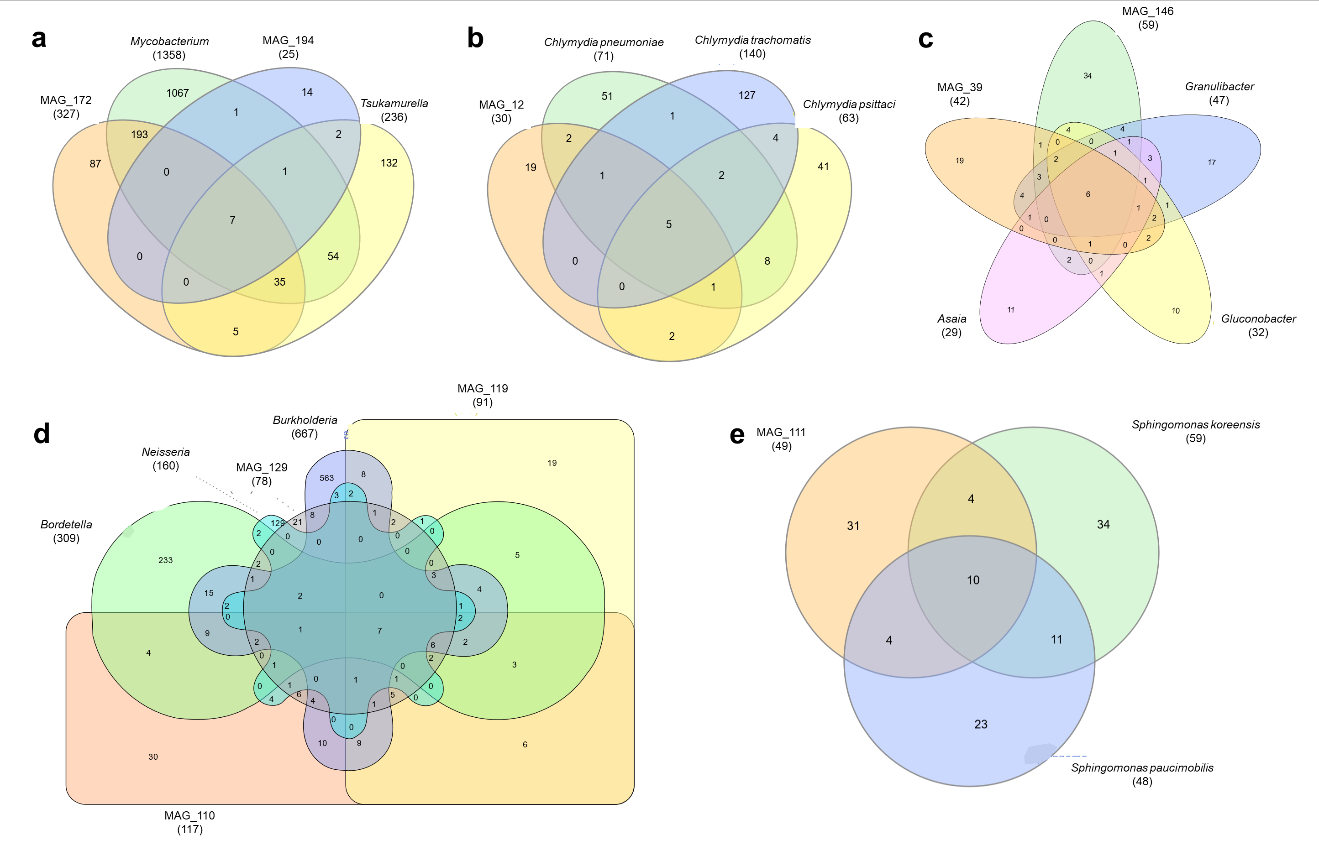
**

**Fig. S4.** Venn diagrams showing the numbers of shared and unique virulence genes detected in clinical and environmental bog isolate genomes. The graph was generated using InteractiVenn [61].

**Supplementary Tables**

**Table S1**. Description of vegetation field plots regarding habitat characteristics and plant coverage per plot.

| Location | Sample ID* | Habitat characteristic by indicator species^#^ | | | | | Plant species | % of plant cover per plot |
| --- | --- | --- | --- | --- | --- | --- | --- | --- |
|  |  | Soil reaction | Nutrient value | Light value | Moisture value | Temperature value |  |  |
| Rotmoos | MS1.1** | 1.37 | 1.69 | 8.07 | 6.57 | 2.98 | *Pleurozium schreberi* | 3 |
|  | MS1.2 |  |  |  |  |  | *Sphagnum angustifolium* | 37 |
|  | MS1.3 |  |  |  |  |  | *Vaccinium myrtillus* | 1 |
|  | MS1.4** |  |  |  |  |  | *Calluna vulgaris* | 10 |
|  | MS1.5 |  |  |  |  |  | *Vaccinium oxycoccos* | 3 |
|  | MS1.6 |  |  |  |  |  | *Pinus mugo* | 0.2 |
|  | MS1.7** |  |  |  |  |  | *Sphagnum fuscum* | 10 |
|  | MS1.8** |  |  |  |  |  | *Andromeda polifolia* | 3 |
|  | MS1.9** |  |  |  |  |  | *Sphagnum magellanicum* | 62 |
|  | MS1.10 |  |  |  |  |  | *Polytrichum strictum* | 5 |
|  | MS1.11** |  |  |  |  |  | *Eriophorum vaginatum* | 10 |
| Pürgschachen Moor | MS1.4** | 1.50 | 1.90 | 7.97 | 6.55 | 2.98 | *Calluna vulgaris* | 20 |
|  | MS1.9** |  |  |  |  |  | *Sphagnum magellanicum* | 37 |
|  | MS1.7** |  |  |  |  |  | *Sphagnum fuscum* | 10 |
|  | MS4.5 |  |  |  |  |  | *Mylia anomala* | 3 |
|  | MS1.1** |  |  |  |  |  | *Pleurozium schreberi* | 3 |
|  | MS1.8** |  |  |  |  |  | *Andromeda polifolia* | 1 |
|  | MS1.11** |  |  |  |  |  | *Eriophorum vaginatum* | 10 |

*These are representative samples from a larger experimental design explained in detail by Taffner et al. 2018 [27].

** Plant samples were pooled from multiple individuals prior to sequencing.

^#^Habitat characteristics are expressed as average Ellenberg’s indicator values for vascular plant and bryophyte species. Numbers indicate properties of the habitat along ecological gradients (soil reaction: 1=extremely acidic, 9=calcareous; nutrient value: 1=extremely nutrient poor, 9=extremely nutrient rich; light exposure: 1=deep shadowed, 9=full light exposed; moisture: 1=extremely dry, 9=extremely wet; temperature: 1=extremely cold, 9=extremely warm).

**Table S2.** List of the 12 plant species that were included to represent the vegetation of a *Sphagnum*-dominated bog ecosystem.

| Sample ID | Species | Type | No. of reads | No. of bacterial reads^*^ | Number of reads assigned to N cycle genes^#^ |
| --- | --- | --- | --- | --- | --- |
| MS1_1 | *Pleurozium schreberi* | Bryophytes | 35,427,233 | 22,546,737 | 24,314 |
| MS1_10 | *Polytrichum strictum* | Bryophytes | 31,021,698 | 23,360,541 | 24,327 |
| MS1_2 | *Sphagnum angustifolium* | Bryophytes | 38,808,131 | 27,927,721 | 36,098 |
| MS1_7 | *Sphagnum fuscum* | Bryophytes | 32,481,892 | 20,858,087 | 17,886 |
| MS1_9 | *Sphagnum magellanicum* | Bryophytes | 29,205,663 | 15,932,875 | 16,078 |
| MS4_5 | *Mylia anomala* | Bryophytes | 25,251,155 | 17,964,766 | 15,806 |
| MS1_3 | *Vaccinium myrtillus* | Vascular plant | 37,240,869 | 17,350,942 | 23,180 |
| MS1_4 | *Calluna vulgaris* | Vascular plant | 22,743,173 | 14,506,084 | 15,953 |
| MS1_5 | *Vaccinium oxycoccus* | Vascular plant | 28,315,816 | 13,182,898 | 14,754 |
| MS1_8 | *Andromeda polifolia* | Vascular plant | 34,343,126 | 15,570,031 | 17,777 |
| MS1_11 | *Eriophorum vaginatum* | Vascular plant | 27,093,667 | 19,393,284 | 21,915 |
| MS1_6 | *Pinus mugo* | Vascular plant | 40,915,817 | 8,563057 | 6,417 |

^*^ Classification according to eggNOG database

^#^ Classification according to NCycDB database

**Table S3.** Detailed taxonomical classification, source of origin and NCBI accessions of clinical and environmental isolate genomes.

| Isolates name | Source of origin | NCBI accession number |
| --- | --- | --- |
| *Mycobacterium simiae* | Human | GCA_000455305.1. |
| *Mycobacterium simiae* | Human | AP022568.1 |
| *Mycobacterium tuberculosis* | Human | WMCK00000000 |
| *Mycobacterium xenopi* | Human | AP022314. |
| *Mycobacterium tuberculosis* | Human | WFLD00000000 |
| *Mycobacterium ulcerans* | Human | ERS3388536 |
| *Mycobacterium setense* | Human | OEJY01000000. |
| *Mycobacterium shimoidei* | Human | UEGW00000000. |
| *Mycobacterium tuberculosis* | Human | SDOF00000000 |
| *Tsukamurella sputi* | Human | VIGV00000000.1 |
| *Tsukamurella asaccharolytica* | Human | VIGW00000000.1 |
| *Tsukamurella conjunctivitidis* | Human | VIGX01000000 |
| *Tsukamurella pulmonis* | Human | LSRH00000000.1 |
| *Tsukamurella tyrosinosolvens* | Human | LSRJ00000000.1 |
| *Tsukamurella columbiensis* | Human | JABARZ000000000 |
| *Tsukamurella pulmonis* | Human | QQQF00000000.1 |
| *Tsukamurella tyrosinosolvens* | Human | QPKD00000000.1 |
| *Chlamydia trachomatis* | Human | CP006945 |
| *Chlamydia trachomatis* | Human | BCAM01000001 |
| *Chlamydia trachomatis* | Human | CAAKND000000000.1 |
| *Chlamydia trachomatis* | Human | CP002024 |
| *Chlamydophila psittaci* | Human | NC_017290.1 |
| *Chlamydia pneumoniae* | Human | NC_000922.1 |
| *Chlamydia pneumoniae* | Human | NC_005043.1 |
| *Chlamydophila pneumoniae* | Human | /AE002161 |
| *Chlamydophila pneumoniae* | Human | BA000008 |
| *Sphingomonas paucimobilis* | Human | AP023323.1 |
| *Sphingomonas koreensis* | Human | CP018820.1 |
| *Sphingomonas wittichii* | Environment | NC_009511.1 |
| *Sphingomonas sanxanigenens* | Environment | CP006644.1 |
| *Sphingomonas paucimobilis* | Environment | JABEOU000000000.1 |
| *Sphingomonas sanguinis* | Environment | JABEOW000000000.1 |
| *Sphingomonas sanguinis* | Environment | JABEOV000000000.1 |
| *Sphingomonas sp.* | Environment | CP029985 |
| *Sphingomonas sp.* | Environment | AFGG00000000 |
| *Granulibacter bethesdensis* | Human | CP000394 |
| *Granulibacter bethesdensis* | Human | CP003182.2 |
| *Asaia bogorensis* | Human | BJVS01000000 |
| *Granulibacter bethesdensis* | Human | CP018195.1 |
| *Granulibacter bethesdensis* | Human | CP018191.1 |
| *Granulibacter bethesdensis* | Human | CP018194.1 |
| *Gluconobacter morbifer* | Animal | AGQV00000000. |
| *Granulibacter bethesdensis* | Human | CP018192.1 |
| *Burkholderia contaminans* | Human | LASD00000000 |
| *Burkholderia contaminans* | Human | LASC00000000 |
| *Burkholderia cepacia* | Human | CP011301 |
| *Burkholderia pseudomallei* | Human | LWRR00000000. |
| *Burkholderia cenocepacia* | Human | CP053300.1 CP053302.1 |
| *Neisseria gonorrhoeae* | Human | AP019853 |
| *Neisseria gonorrhoeae* | Human | CP034032 |
| *Bordetella holmesii* | Human | CP007494 |
| *Bordetella holmesii* | Human | JDSC00000000 |
| *Bordetella pertussis* | Human | CP031788 |

**Table S4.** List of the KEGG orthologs that were significantly enriched between bryophytes and vascular plants.

| KEGG ORTHOLOGY | log_2_FC | P_adjusted_ values | Details |
| --- | --- | --- | --- |
| K12055 | -3.0 | 0.012 | Enriched in bryophytes |
| K11950 | -2.4 | 0.016 | Enriched in bryophytes |
| K20424 | -2.4 | 0.011 | Enriched in bryophytes |
| K21601 | -1.9 | 0.014 | Enriched in bryophytes |
| K16153 | -1.9 | 0.001 | Enriched in bryophytes |
| K21960 | -1.8 | 0.010 | Enriched in bryophytes |
| K18376 | -1.8 | 0.042 | Enriched in bryophytes |
| K15750 | -1.8 | 0.003 | Enriched in bryophytes |
| K20148 | -1.7 | 0.011 | Enriched in bryophytes |
| K15751 | -1.7 | 0.011 | Enriched in bryophytes |
| K19139 | -1.6 | 0.001 | Enriched in bryophytes |
| K07450 | -1.6 | 0.015 | Enriched in bryophytes |
| K22305 | -1.5 | 0.004 | Enriched in bryophytes |
| K00462 | -1.5 | 0.007 | Enriched in bryophytes |
| K14748 | -1.5 | 0.005 | Enriched in bryophytes |
| K16233 | -1.5 | 0.011 | Enriched in bryophytes |
| K17506 | -1.5 | 0.075 | Enriched in bryophytes |
| K11021 | -1.5 | 0.000 | Enriched in bryophytes |
| K07535 | -1.5 | 0.017 | Enriched in bryophytes |
| K12212 | -1.5 | 0.061 | Enriched in bryophytes |
| K14585 | -1.4 | 0.030 | Enriched in bryophytes |
| K05919 | -1.4 | 0.017 | Enriched in bryophytes |
| K03268 | -1.4 | 0.004 | Enriched in bryophytes |
| K15534 | -1.4 | 0.047 | Enriched in bryophytes |
| K18092 | -1.3 | 0.018 | Enriched in bryophytes |
| K07646 | -1.3 | 0.035 | Enriched in bryophytes |
| K03095 | -1.3 | 0.016 | Enriched in bryophytes |
| K07099 | -1.3 | 0.089 | Enriched in bryophytes |
| K03589 | -1.3 | 0.002 | Enriched in bryophytes |
| K07584 | -1.3 | 0.018 | Enriched in bryophytes |
| K14580 | -1.3 | 0.028 | Enriched in bryophytes |
| K04618 | -1.3 | 0.011 | Enriched in bryophytes |
| K02334 | -1.3 | 0.004 | Enriched in bryophytes |
| K07790 | -1.2 | 0.008 | Enriched in bryophytes |
| K09820 | -1.2 | 0.011 | Enriched in bryophytes |
| K18014 | -1.2 | 0.041 | Enriched in bryophytes |
| K14161 | -1.2 | 0.018 | Enriched in bryophytes |
| K05708 | -1.2 | 0.017 | Enriched in bryophytes |
| K07016 | -1.2 | 0.016 | Enriched in bryophytes |
| K07485 | -1.2 | 0.001 | Enriched in bryophytes |
| K09822 | -1.2 | 0.001 | Enriched in bryophytes |
| K06335 | -1.2 | 0.027 | Enriched in bryophytes |
| K08603 | -1.2 | 0.018 | Enriched in bryophytes |
| K22187 | -1.2 | 0.008 | Enriched in bryophytes |
| K02761 | -1.1 | 0.015 | Enriched in bryophytes |
| K13276 | -1.1 | 0.020 | Enriched in bryophytes |
| K09002 | -1.1 | 0.027 | Enriched in bryophytes |
| K07469 | -1.1 | 0.008 | Enriched in bryophytes |
| K18011 | -1.1 | 0.029 | Enriched in bryophytes |
| K09000 | -1.1 | 0.058 | Enriched in bryophytes |
| K04028 | -1.1 | 0.018 | Enriched in bryophytes |
| K00166 | -1.1 | 0.007 | Enriched in bryophytes |
| K12513 | -1.1 | 0.031 | Enriched in bryophytes |
| K09968 | -1.1 | 0.073 | Enriched in bryophytes |
| K03332 | -1.1 | 0.019 | Enriched in bryophytes |
| K14330 | -1.1 | 0.014 | Enriched in bryophytes |
| K12205 | -1.1 | 0.069 | Enriched in bryophytes |
| K18195 | -1.1 | 0.001 | Enriched in bryophytes |
| K19138 | -1.1 | 0.044 | Enriched in bryophytes |
| K14061 | -1.1 | 0.057 | Enriched in bryophytes |
| K16388 | -1.0 | 0.043 | Enriched in bryophytes |
| K02007 | -1.0 | 0.012 | Enriched in bryophytes |
| K08076 | -1.0 | 0.044 | Enriched in bryophytes |
| K12547 | -1.0 | 0.064 | Enriched in bryophytes |
| K14682 | -1.0 | 0.098 | Enriched in bryophytes |
| K13311 | -1.0 | 0.063 | Enriched in bryophytes |
| K07066 | -1.0 | 0.044 | Enriched in bryophytes |
| K09859 | -1.0 | 0.086 | Enriched in bryophytes |
| K01121 | -1.0 | 0.012 | Enriched in bryophytes |
| K18012 | -1.0 | 0.029 | Enriched in bryophytes |
| K09951 | -1.0 | 0.011 | Enriched in bryophytes |
| K03738 | -1.0 | 0.094 | Enriched in bryophytes |
| K02361 | -1.0 | 0.023 | Enriched in bryophytes |
| K12956 | -1.0 | 0.043 | Enriched in bryophytes |
| K07273 | 1.0 | 0.071 | Enriched in vascular plants |
| K10708 | 1.0 | 0.098 | Enriched in vascular plants |
| K07013 | 1.0 | 0.097 | Enriched in vascular plants |
| K07345 | 1.0 | 0.092 | Enriched in vascular plants |
| K12980 | 1.0 | 0.086 | Enriched in vascular plants |
| K07653 | 1.0 | 0.004 | Enriched in vascular plants |
| K12541 | 1.0 | 0.099 | Enriched in vascular plants |
| K11211 | 1.0 | 0.071 | Enriched in vascular plants |
| K13933 | 1.0 | 0.040 | Enriched in vascular plants |
| K21472 | 1.0 | 0.008 | Enriched in vascular plants |
| K04093 | 1.0 | 0.010 | Enriched in vascular plants |
| K00035 | 1.1 | 0.003 | Enriched in vascular plants |
| K00009 | 1.1 | 0.000 | Enriched in vascular plants |
| K21104 | 1.1 | 0.070 | Enriched in vascular plants |
| K07498 | 1.1 | 0.012 | Enriched in vascular plants |
| K03203 | 1.1 | 0.010 | Enriched in vascular plants |
| K03776 | 1.1 | 0.054 | Enriched in vascular plants |
| K13929 | 1.1 | 0.001 | Enriched in vascular plants |
| K12688 | 1.1 | 0.065 | Enriched in vascular plants |
| K07518 | 1.1 | 0.073 | Enriched in vascular plants |
| K00947 | 1.1 | 0.047 | Enriched in vascular plants |
| K10545 | 1.1 | 0.006 | Enriched in vascular plants |
| K01004 | 1.1 | 0.060 | Enriched in vascular plants |
| K10119 | 1.1 | 0.040 | Enriched in vascular plants |
| K13930 | 1.1 | 0.019 | Enriched in vascular plants |
| K06213 | 1.1 | 0.022 | Enriched in vascular plants |
| K12428 | 1.1 | 0.085 | Enriched in vascular plants |
| K09988 | 1.1 | 0.061 | Enriched in vascular plants |
| K13408 | 1.1 | 0.094 | Enriched in vascular plants |
| K00957 | 1.1 | 0.024 | Enriched in vascular plants |
| K11105 | 1.1 | 0.041 | Enriched in vascular plants |
| K09190 | 1.1 | 0.034 | Enriched in vascular plants |
| K03795 | 1.1 | 0.021 | Enriched in vascular plants |
| K18900 | 1.1 | 0.008 | Enriched in vascular plants |
| K01451 | 1.1 | 0.063 | Enriched in vascular plants |
| K18288 | 1.1 | 0.031 | Enriched in vascular plants |
| K22108 | 1.1 | 0.046 | Enriched in vascular plants |
| K09758 | 1.1 | 0.008 | Enriched in vascular plants |
| K19587 | 1.1 | 0.073 | Enriched in vascular plants |
| K18302 | 1.1 | 0.018 | Enriched in vascular plants |
| K03523 | 1.1 | 0.050 | Enriched in vascular plants |
| K17317 | 1.1 | 0.066 | Enriched in vascular plants |
| K05841 | 1.1 | 0.015 | Enriched in vascular plants |
| K17324 | 1.1 | 0.017 | Enriched in vascular plants |
| K05782 | 1.1 | 0.019 | Enriched in vascular plants |
| K19736 | 1.1 | 0.077 | Enriched in vascular plants |
| K18697 | 1.1 | 0.027 | Enriched in vascular plants |
| K02623 | 1.2 | 0.018 | Enriched in vascular plants |
| K20382 | 1.2 | 0.063 | Enriched in vascular plants |
| K16783 | 1.2 | 0.060 | Enriched in vascular plants |
| K13931 | 1.2 | 0.015 | Enriched in vascular plants |
| K01031 | 1.2 | 0.054 | Enriched in vascular plants |
| K00545 | 1.2 | 0.092 | Enriched in vascular plants |
| K13012 | 1.2 | 0.064 | Enriched in vascular plants |
| K02424 | 1.2 | 0.087 | Enriched in vascular plants |
| K02625 | 1.2 | 0.027 | Enriched in vascular plants |
| K02015 | 1.2 | 0.047 | Enriched in vascular plants |
| K09131 | 1.2 | 0.026 | Enriched in vascular plants |
| K02552 | 1.2 | 0.032 | Enriched in vascular plants |
| K10125 | 1.2 | 0.026 | Enriched in vascular plants |
| K12955 | 1.2 | 0.098 | Enriched in vascular plants |
| K10234 | 1.2 | 0.013 | Enriched in vascular plants |
| K21568 | 1.2 | 0.083 | Enriched in vascular plants |
| K11055 | 1.2 | 0.034 | Enriched in vascular plants |
| K10555 | 1.2 | 0.009 | Enriched in vascular plants |
| K07673 | 1.2 | 0.079 | Enriched in vascular plants |
| K03897 | 1.2 | 0.064 | Enriched in vascular plants |
| K00098 | 1.2 | 0.010 | Enriched in vascular plants |
| K16516 | 1.2 | 0.075 | Enriched in vascular plants |
| K11386 | 1.2 | 0.085 | Enriched in vascular plants |
| K22024 | 1.2 | 0.011 | Enriched in vascular plants |
| K02588 | 1.2 | 0.089 | Enriched in vascular plants |
| K11925 | 1.2 | 0.066 | Enriched in vascular plants |
| K08156 | 1.2 | 0.066 | Enriched in vascular plants |
| K04641 | 1.2 | 0.034 | Enriched in vascular plants |
| K13060 | 1.2 | 0.014 | Enriched in vascular plants |
| K20431 | 1.2 | 0.063 | Enriched in vascular plants |
| K10553 | 1.3 | 0.017 | Enriched in vascular plants |
| K03449 | 1.3 | 0.045 | Enriched in vascular plants |
| K07691 | 1.3 | 0.048 | Enriched in vascular plants |
| K00424 | 1.3 | 0.092 | Enriched in vascular plants |
| K17323 | 1.3 | 0.010 | Enriched in vascular plants |
| K14055 | 1.3 | 0.031 | Enriched in vascular plants |
| K01792 | 1.3 | 0.010 | Enriched in vascular plants |
| K02591 | 1.3 | 0.062 | Enriched in vascular plants |
| K21119 | 1.3 | 0.045 | Enriched in vascular plants |
| K00039 | 1.3 | 0.012 | Enriched in vascular plants |
| K21022 | 1.3 | 0.023 | Enriched in vascular plants |
| K21200 | 1.3 | 0.055 | Enriched in vascular plants |
| K04784 | 1.3 | 0.009 | Enriched in vascular plants |
| K03672 | 1.3 | 0.025 | Enriched in vascular plants |
| K10547 | 1.3 | 0.011 | Enriched in vascular plants |
| K13409 | 1.3 | 0.081 | Enriched in vascular plants |
| K10227 | 1.3 | 0.028 | Enriched in vascular plants |
| K03477 | 1.3 | 0.006 | Enriched in vascular plants |
| K03745 | 1.3 | 0.034 | Enriched in vascular plants |
| K08095 | 1.3 | 0.076 | Enriched in vascular plants |
| K07165 | 1.3 | 0.000 | Enriched in vascular plants |
| K00370 | 1.3 | 0.024 | Enriched in vascular plants |
| K04789 | 1.3 | 0.054 | Enriched in vascular plants |
| K14728 | 1.3 | 0.004 | Enriched in vascular plants |
| K00998 | 1.3 | 0.014 | Enriched in vascular plants |
| K02431 | 1.3 | 0.045 | Enriched in vascular plants |
| K15270 | 1.4 | 0.019 | Enriched in vascular plants |
| K00371 | 1.4 | 0.027 | Enriched in vascular plants |
| K11628 | 1.4 | 0.027 | Enriched in vascular plants |
| K02444 | 1.4 | 0.000 | Enriched in vascular plants |
| K07168 | 1.4 | 0.024 | Enriched in vascular plants |
| K13794 | 1.4 | 0.027 | Enriched in vascular plants |
| K06857 | 1.4 | 0.010 | Enriched in vascular plants |
| K10539 | 1.4 | 0.027 | Enriched in vascular plants |
| K18700 | 1.4 | 0.020 | Enriched in vascular plants |
| K22144 | 1.4 | 0.005 | Enriched in vascular plants |
| K03760 | 1.4 | 0.012 | Enriched in vascular plants |
| K21090 | 1.4 | 0.044 | Enriched in vascular plants |
| K10021 | 1.4 | 0.031 | Enriched in vascular plants |
| K03777 | 1.4 | 0.028 | Enriched in vascular plants |
| K18120 | 1.4 | 0.030 | Enriched in vascular plants |
| K07029 | 1.4 | 0.029 | Enriched in vascular plants |
| K06145 | 1.4 | 0.009 | Enriched in vascular plants |
| K12989 | 1.5 | 0.010 | Enriched in vascular plants |
| K08312 | 1.5 | 0.014 | Enriched in vascular plants |
| K21298 | 1.5 | 0.016 | Enriched in vascular plants |
| K18642 | 1.5 | 0.010 | Enriched in vascular plants |
| K10551 | 1.5 | 0.026 | Enriched in vascular plants |
| K12432 | 1.5 | 0.005 | Enriched in vascular plants |
| K07543 | 1.5 | 0.017 | Enriched in vascular plants |
| K19246 | 1.5 | 0.031 | Enriched in vascular plants |
| K01757 | 1.5 | 0.020 | Enriched in vascular plants |
| K10009 | 1.5 | 0.041 | Enriched in vascular plants |
| K02586 | 1.5 | 0.026 | Enriched in vascular plants |
| K10538 | 1.5 | 0.028 | Enriched in vascular plants |
| K05548 | 1.5 | 0.002 | Enriched in vascular plants |
| K00886 | 1.5 | 0.089 | Enriched in vascular plants |
| K11216 | 1.5 | 0.001 | Enriched in vascular plants |
| K18851 | 1.5 | 0.015 | Enriched in vascular plants |
| K03151 | 1.5 | 0.011 | Enriched in vascular plants |
| K14699 | 1.6 | 0.031 | Enriched in vascular plants |
| K18996 | 1.6 | 0.001 | Enriched in vascular plants |
| K17737 | 1.6 | 0.034 | Enriched in vascular plants |
| K10020 | 1.6 | 0.029 | Enriched in vascular plants |
| K21756 | 1.6 | 0.012 | Enriched in vascular plants |
| K21375 | 1.6 | 0.016 | Enriched in vascular plants |
| K16087 | 1.6 | 0.009 | Enriched in vascular plants |
| K13928 | 1.6 | 0.055 | Enriched in vascular plants |
| K00875 | 1.6 | 0.011 | Enriched in vascular plants |
| K13281 | 1.6 | 0.012 | Enriched in vascular plants |
| K16053 | 1.6 | 0.022 | Enriched in vascular plants |
| K03818 | 1.6 | 0.079 | Enriched in vascular plants |
| K10117 | 1.6 | 0.004 | Enriched in vascular plants |
| K10126 | 1.7 | 0.034 | Enriched in vascular plants |
| K03721 | 1.7 | 0.044 | Enriched in vascular plants |
| K20972 | 1.7 | 0.089 | Enriched in vascular plants |
| K19337 | 1.7 | 0.040 | Enriched in vascular plants |
| K07782 | 1.7 | 0.015 | Enriched in vascular plants |
| K05878 | 1.7 | 0.017 | Enriched in vascular plants |
| K08221 | 1.7 | 0.008 | Enriched in vascular plants |
| K10111 | 1.7 | 0.011 | Enriched in vascular plants |
| K02002 | 1.7 | 0.023 | Enriched in vascular plants |
| K21199 | 1.7 | 0.011 | Enriched in vascular plants |
| K02775 | 1.7 | 0.001 | Enriched in vascular plants |
| K12950 | 1.8 | 0.011 | Enriched in vascular plants |
| K10540 | 1.8 | 0.015 | Enriched in vascular plants |
| K10564 | 1.8 | 0.010 | Enriched in vascular plants |
| K16135 | 1.8 | 0.034 | Enriched in vascular plants |
| K00862 | 1.8 | 0.004 | Enriched in vascular plants |
| K02585 | 1.8 | 0.007 | Enriched in vascular plants |
| K00895 | 1.8 | 0.068 | Enriched in vascular plants |
| K17204 | 1.8 | 0.002 | Enriched in vascular plants |
| K19731 | 1.9 | 0.026 | Enriched in vascular plants |
| K06605 | 1.9 | 0.013 | Enriched in vascular plants |
| K10118 | 1.9 | 0.009 | Enriched in vascular plants |
| K10018 | 1.9 | 0.011 | Enriched in vascular plants |
| K09794 | 1.9 | 0.006 | Enriched in vascular plants |
| K02288 | 1.9 | 0.006 | Enriched in vascular plants |
| K00413 | 1.9 | 0.062 | Enriched in vascular plants |
| K07093 | 1.9 | 0.049 | Enriched in vascular plants |
| K14028 | 1.9 | 0.000 | Enriched in vascular plants |
| K04792 | 1.9 | 0.012 | Enriched in vascular plants |
| K05851 | 1.9 | 0.018 | Enriched in vascular plants |
| K16228 | 1.9 | 0.002 | Enriched in vascular plants |
| K18990 | 2.0 | 0.014 | Enriched in vascular plants |
| K02567 | 2.0 | 0.004 | Enriched in vascular plants |
| K02168 | 2.0 | 0.018 | Enriched in vascular plants |
| K01085 | 2.0 | 0.031 | Enriched in vascular plants |
| K13255 | 2.1 | 0.011 | Enriched in vascular plants |
| K06609 | 2.1 | 0.074 | Enriched in vascular plants |
| K17938 | 2.1 | 0.008 | Enriched in vascular plants |
| K09781 | 2.1 | 0.007 | Enriched in vascular plants |
| K10019 | 2.1 | 0.011 | Enriched in vascular plants |
| K07401 | 2.1 | 0.011 | Enriched in vascular plants |
| K16088 | 2.2 | 0.000 | Enriched in vascular plants |
| K07231 | 2.3 | 0.032 | Enriched in vascular plants |
| K19733 | 2.3 | 0.002 | Enriched in vascular plants |
| K09799 | 2.4 | 0.035 | Enriched in vascular plants |
| K03445 | 2.4 | 0.011 | Enriched in vascular plants |
| K03229 | 2.4 | 0.024 | Enriched in vascular plants |
| K03894 | 2.4 | 0.001 | Enriched in vascular plants |
| K18652 | 2.5 | 0.025 | Enriched in vascular plants |
| K06193 | 2.6 | 0.001 | Enriched in vascular plants |
| K11045 | 2.6 | 0.049 | Enriched in vascular plants |
| K01908 | 2.9 | 0.003 | Enriched in vascular plants |
| K18982 | 2.9 | 0.010 | Enriched in vascular plants |
| K18093 | 2.9 | 0.004 | Enriched in vascular plants |
| K17207 | 3.0 | 0.005 | Enriched in vascular plants |
| K05559 | 3.0 | 0.004 | Enriched in vascular plants |
| K15054 | 3.0 | 0.001 | Enriched in vascular plants |
| K11059 | 3.2 | 0.008 | Enriched in vascular plants |
| K03469 | 3.3 | 0.004 | Enriched in vascular plants |
| K07655 | 3.4 | 0.003 | Enriched in vascular plants |
| K17205 | 3.5 | 0.003 | Enriched in vascular plants |
| K20268 | 3.7 | 0.001 | Enriched in vascular plants |
| K20325 | 4.1 | 0.001 | Enriched in vascular plants |
| K08693 | 4.2 | 0.001 | Enriched in vascular plants |
| K05358 | 4.2 | 0.002 | Enriched in vascular plants |
| K19290 | 4.4 | 0.000 | Enriched in vascular plants |

**Table S5.** List of the pathways and genes involved in nitrogen cycling that were significantly enriched in either bryophytes or vascular plants.

| Pathways/Genes | log_2_FC | P_adjusted_ values | Details |
| --- | --- | --- | --- |
| Pathways |  |  |  |
| Nitrogen_fixation | -0.7 | 0.003 | Enriched in bryophytes |
| Denitrification | -0.4 | 0.001 | Enriched in bryophytes |
| Others | 2.7 | <0.001 | Enriched in vascular |
|  |  |  |  |
| Genes |  |  |  |
| *nifH* | -1.2 | <0.001 | Enriched in bryophytes |
| *GLT1* | 1.3 | <0.001 | Enriched in vascular plants |
| *NR* | 1.3 | 0.030 | Enriched in vascular plants |
| *narH* | 1.5 | 0.025 | Enriched in vascular plants |
| *narZ* | 1.7 | 0.049 | Enriched in vascular plants |
| *pmoB* | 2.7 | 0.014 | Enriched in vascular plants |
| *pmoC* | 3.5 | 0.001 | Enriched in vascular plants |

**Table S6.** Detailed taxonomic classification, completeness, contamination values, and genome sizes of bacterial MAGs.

| MAG ID | Taxonomic classification | Completeness | Contamination | Genome size (bp) |
| --- | --- | --- | --- | --- |
| MAG_172 | *Mycobacterium* (*Actinobacteria*) | 95.1 | 9.2 | 6,564,149 |
| MAG_194 | *Tsukamurellaceae* (*Actinobacteria*) | 91.4 | 6.4 | 4,992,893 |
| MAG_24 | *Actinobacteria* (*Actinobacteria*) | 87.1 | 1.9 | 2,597,317 |
| MAG_9 | *Actinobacteria* bacterium 21-64-8 (*Actinobacteria*) | 95.7 | 1.3 | 1,972,358 |
| MAG_142 | *Actinobacteria* bacterium (*Actinobacteria*) | 89.0 | 3.7 | 2,655,961 |
| MAG_22 | unclassified *Candidatus Eremiobacteraeota* | 91.5 | 2.9 | 3,260,124 |
| MAG_12 | *Chlamydiae* (*Chlamydiae*) | 92.4 | 1.4 | 2,092,162 |
| MAG_10 | unclassified *Verrucomicrobia* (*Verrucomicrobia*) | 88.1 | 6.4 | 2,926,007 |
| MAG_178 | unclassified Verrucomicrobia (*Verrucomicrobia*) | 88.0 | 3.0 | 2,882,424 |
| MAG_134 | unclassified *Acidobacteria* (*Acidobacteria*) | 93.6 | 4.7 | 5,607,130 |
| MAG_201 | unclassified *Bryobacterales* (*Acidobacteria*) | 96.8 | 3.0 | 5,452,513 |
| MAG_144 | *Acidisarcina (Acidobacteria*) | 83.3 | 7.0 | 4,296,633 |
| MAG_136 | *Granulicella* (*Acidobacteria*) | 96.4 | 7.0 | 4,133,772 |
| MAG_57 | *Granulicella* (*Acidobacteria*) | 97.2 | 5.6 | 3,678,110 |
| MAG_202 | *Granulicella* (*Acidobacteria*) | 83.2 | 0.9 | 3,824,088 |
| MAG_63 | *Bacteroidetes* (*Bacteroidetes*) | 88.3 | 2.1 | 3,137,154 |
| MAG_200 | *Dinghuibacter* (*Bacteroidetes*) | 96.7 | 1.9 | 5,352,509 |
| MAG_154 | *Rhizobium* (*Proteobacteria*) | 97.6 | 2.1 | 4,858,929 |
| MAG_111 | *Sphingomonadaceae* (*Proteobacteria*) | 88.4 | 6.1 | 4,090,219 |
| MAG_146 | *Rhodospirillales* (*Proteobacteria*) | 80.6 | 6.7 | 3,343,448 |
| MAG_39 | *Rhodospirillales* (*Proteobacteria*) | 96.1 | 3.7 | 4,289,667 |
| MAG_2 | *Gammaproteobacteria* (*Proteobacteria*) | 97.0 | 0.6 | 5,517,870 |
| MAG_99 | *Gammaproteobacteria* (*Proteobacteria*) | 93.0 | 5.7 | 2,944,536 |
| MAG_MB_405 | *Gammaproteobacteria* (*Proteobacteria*) | 86.7 | 7.5 | 1,828,578 |
| MAG_93 | *unclassified Dyella* (*Proteobacteria*) | 92.6 | 6.2 | 4,528,035 |
| MAG_119 | *Betaproteobacteria* (*Proteobacteria*) | 96.2 | 3.3 | 3,466,724 |
| MAG_129 | *Betaproteobacteria* (*Proteobacteria*) | 96.6 | 8.0 | 3,708,935 |
| MAG_110 | *Betaproteobacteria* (*Proteobacteria*) | 92.8 | 1.7 | 4,334,065 |

| MAG ID | Taxonomy classsification | Bryophytes^#^ | | | | | | Vascular plants^#^ | | | | | | P value |
| --- | --- | --- | --- | --- | --- | --- | --- | --- | --- | --- | --- | --- | --- | --- |
|  |  | MS1_9 | MS1_2 | MS1_7 | MS1_1 | MS1_10 | MS4_5 | MS1_3 | MS1_4 | MS1_5 | MS1_8 | MS1_11 | MS1_6 |  |
| MAG_172 | *Mycobacterium* | 6.9 | 6.1 | 6.3 | 9.5 | 7.5 | 2.9 | 4.5 | 9.4 | 8.4 | 18.6 | 9.3 | 15.4 | 0.130 |
| MAG_194 | *Tsukamurellaceae* | 7.1 | 6.0 | 7.0 | 7.6 | 17.7 | 1.0 | 3.7 | 8.6 | 7.3 | 7.1 | 8.2 | 5.9 | 0.940 |
| MAG_24 | *Actinobacteria* | 13.8 | 12.3 | 13.5 | 25.3 | 7.7 | 3.1 | 8.3 | 17.4 | 15.7 | 13.4 | 16.6 | 12.4 | 0.480 |
| MAG_9 | *Actinobacteria* bacterium 21-64-8 | 8.4 | 3.4 | 12.7 | 3.9 | 1.3 | 5.4 | 2.6 | 4.9 | 4.5 | 4.5 | 4.7 | 4.6 | 0.820 |
| MAG_142 | *Actinobacteria* bacterium | 10.7 | 6.9 | 21.9 | 8.4 | 4.3 | 12.9 | 5.4 | 9.3 | 8.6 | 8.4 | 9.7 | 9.1 | 0.820 |
| MAG_22 | *Candidatus Eremiobacteraeota* | 15.9 | 12.0 | 21.4 | 22.5 | 9.9 | 10.7 | 4.9 | 13.1 | 13.4 | 11.5 | 11.8 | 8.9 | 0.240 |
| MAG_12 | *Chlamydiae* | 16.7 | 5.5 | 4.6 | 2.2 | 2.0 | 0.8 | 3.7 | 3.6 | 4.8 | 5.0 | 2.1 | 11.8 | 0.700 |
| MAG_10 | unclassified *Verrucomicrobia* | 10.5 | 11.9 | 9.3 | 23.5 | 12.9 | 6.1 | 4.3 | 12.9 | 10.2 | 9.9 | 11.4 | 8.8 | 0.390 |
| **MAG_178** | **unclassified *Verrucomicrobia*** | 8.4 | 14.9 | 7.6 | 9.5 | 14.4 | 10.3 | 4.2 | 9.0 | 7.1 | 8.5 | 8.2 | 8.1 | 0.065* |
| MAG_134 | *unclassified Acidobacteria* | 8.0 | 5.7 | 5.5 | 3.5 | 2.6 | 2.5 | 3.5 | 4.9 | 6.9 | 6.7 | 5.4 | 6.9 | 0.480 |
| **MAG_201** | **unclassified *Bryobacterales*** | 14.5 | 5.8 | 20.4 | 4.0 | 22.0 | 20.7 | 2.6 | 6.0 | 4.9 | 5.6 | 5.6 | 6.6 | 0.093* |
| MAG_144 | *Acidisarcina* | 11.7 | 9.1 | 11.3 | 10.2 | 16.3 | 22.3 | 3.8 | 11.2 | 13.5 | 11.5 | 14.1 | 9.9 | 0.590 |
| MAG_136 | *Granulicella* | 9.7 | 24.5 | 9.4 | 11.2 | 10.2 | 11.2 | 3.3 | 11.2 | 10.2 | 9.5 | 13.1 | 7.5 | 0.390 |
| MAG_57 | *Granulicella* | 4.6 | 4.6 | 4.5 | 15.7 | 5.8 | 4.6 | 2.2 | 5.8 | 5.7 | 5.9 | 7.2 | 6.0 | 0.390 |
| MAG_202 | *Granulicella* | 8.2 | 27.1 | 7.9 | 10.4 | 16.2 | 9.5 | 2.8 | 9.5 | 10.6 | 9.7 | 13.3 | 7.1 | 0.480 |
| MAG_63 | *Bacteroidetes* | 6.7 | 3.9 | 4.8 | 3.2 | 1.1 | 6.7 | 5.7 | 4.3 | 6.8 | 7.1 | 2.9 | 17.7 | 0.240 |
| MAG_200 | *Dinghuibacter* | 2.7 | 2.3 | 2.1 | 2.4 | 9.2 | 0.2 | 1.9 | 2.3 | 8.7 | 4.5 | 1.8 | 6.8 | 0.820 |
| **MAG_154** | ***Rhizobium*** | 7.7 | 6.8 | 7.4 | 7.5 | 7.4 | 5.1 | 84.3 | 10.3 | 9.0 | 8.8 | 9.7 | 7.3 | 0.026* |
| **MAG_111** | ***Sphingomonadaceae*** | 11.1 | 9.6 | 10.7 | 10.9 | 6.2 | 7.4 | 16.5 | 17.9 | 12.3 | 11.7 | 13.2 | 10.1 | 0.015* |
| MAG_146 | *Rhodospirillales* | 22.6 | 23.5 | 23.0 | 27.9 | 17.6 | 35.9 | 11.2 | 27.8 | 24.7 | 21.0 | 24.2 | 19.3 | 0.159 |
| MAG_39 | *Rhodospirillales* | 16.4 | 25.2 | 15.8 | 14.6 | 9.8 | 40.7 | 7.9 | 15.5 | 16.4 | 14.5 | 15.4 | 15.8 | 0.310 |
| **MAG_2** | ***Gammaproteobacteria*** | 5.9 | 5.5 | 5.5 | 5.9 | 1.9 | 1.7 | 24.6 | 7.3 | 6.3 | 6.6 | 8.0 | 6.1 | 0.002* |
| MAG_99 | *Gammaproteobacteria* | 4.1 | 2.4 | 3.9 | 2.5 | 1.4 | 20.5 | 4.1 | 3.1 | 4.9 | 5.5 | 2.6 | 13.1 | 0.240 |
| MAG_MB_405 | *Gammaproteobacteria* | 3.8 | 3.0 | 4.1 | 2.9 | 2.3 | 19.3 | 5.3 | 4.2 | 5.3 | 5.5 | 3.2 | 10.5 | 0.130 |
| **MAG_93** | **unclassified *Dyella*** | 6.1 | 6.3 | 5.2 | 6.6 | 8.1 | 2.3 | 3.0 | 8.8 | 10.3 | 8.7 | 10.9 | 6.8 | 0.065* |
| **MAG_119** | ***Betaproteobacteria*** | 6.2 | 5.4 | 5.6 | 5.5 | 5.5 | 3.7 | 2.9 | 8.0 | 7.2 | 7.3 | 11.3 | 8.1 | 0.065* |
| MAG_129 | *Betaproteobacteria* | 7.6 | 6.4 | 8.7 | 6.5 | 15.3 | 2.3 | 2.9 | 7.5 | 6.8 | 7.0 | 7.3 | 6.0 | 0.590 |
| MAG_110 | *Betaproteobacteria* | 9.3 | 5.1 | 5.7 | 5.1 | 8.4 | 1.9 | 2.9 | 6.6 | 7.8 | 8.4 | 6.2 | 6.9 | 0.159 |

**Table S7.** Abundance estimation of metagenome assembled genomes in each plant sample.

^#^value represents number of mapped reads per kilobase per million reads (RPKM) divided by the metagenomic sample sizes (in millions of reads) and the length of the MAG in kilobases

*Significantly different according to pairwise Wilcox test at P*adjusted* <0.1

**Table S8.** Prevalence of putative virulence factors as predicted for metagenome-assembled genomes (MAGs) using the virulence factor database (VFDB).

| VFDB ID^*^ | Prevalence (%) | Gene | Description |
| --- | --- | --- | --- |
| VFG043573 | 92.9 | *CT396* | molecular chaperone DnaK |
| VFG000077 | 85.7 | *clpP* | ATP-dependent Clp protease proteolytic subunit |
| VFG011430 | 71.4 | *acpXL* | acyl carrier protein |
| VFG038840 | 60.7 | *flmH* | flagellar-related 3-oxoacyl-ACP reductase |
| VFG011414 | 57.1 | *kdsA* | 2-dehydro-3-deoxyphosphooctonate aldolase |
| VFG013197 | 50.0 | *hemB* | Porphobilinogen synthase |
| VFG013618 | 50.0 | *hemL* | glutamate-1-semialdehyde aminotransferase |
| VFG049190 | 46.4 | *KOX_00005* | protein disaggregation chaperone |
| VFG000079 | 46.4 | *clpC* | endopeptidase Clp ATP-binding chain C |
| VFG017989 | 46.4 | *C8J_1346* | hypothetical protein |
| VFG018662 | 42.9 | *eno* | enolase |
| VFG046458 | 39.3 | *tufA* | elongation factor Tu |
| VFG041304 | 39.3 | *lirB* | Dot/Icm type IV secretion system effector LirB |
| VFG039536 | 35.7 | *CBU_1566* | Type IVB secretion system translocated effector |
| VFG045692 | 35.7 | *htpB* | molecular chaperone GroEL |
| VFG002225 | 32.1 | *gmd* | GDP-mannose 4,6-dehydratase |
| VFG047710 | 32.1 | *FN3523_0021* | carbamoyl-phosphate synthase large chain |
| VFG015885 | 32.1 | *cysC1* | adenylylsulfate kinase |
| VFG013531 | 28.6 | *pgi* | glucose-6-phosphate isomerase |
| VFG007659 | 28.6 | *rmlB* | dTDP-glucose 4,6-dehydratase |
| VFG046459 | 28.6 | *Fphi_1039* | elongation factor Tu |
| VFG044389 | 28.6 | *pdtorfF* | putative sulfurylase |
| VFG037990 | 28.6 | *BJAB07104_00096* | putative UDP-glucose 6-dehydrogenase |
| VFG026325 | 28.6 | *gmd-1* | GDP-mannose 4,6-dehydratase |
| VFG016532 | 28.6 | *oppF* | oligopeptide ABC transporter, permease component |
| VFG001867 | 25.0 | *sodB* | superoxide dismutase |
| VFG012103 | 25.0 | *groEL* | chaperonin GroEL |
| VFG049195 | 25.0 | *clpB* | protein disaggregation chaperone |
| VFG006476 | 25.0 | *ureB* | urease |

^*^Only virulence genes that were found in at least seven MAGs (25% of the total number of recovered MAGs) are shown.

**Table S9.** Putative virulence factors that were uniquely present in the MAGs generated from the bog ecosystem when compared to their closely related clinical isolates.

| Comparison | Virulence factor | Description |
| --- | --- | --- |
| MAG_110 vs pathogenic *Betaproteobacteria* (*Bordetella*, *Burkholderia* and *Neisseria*) | Dot/Icm | Secretion system |
| MAG_110 vs pathogenic *Betaproteobacteria* (*Bordetella*, *Burkholderia* and *Neisseria*) | hxc | - |
| MAG_110 vs pathogenic *Betaproteobacteria* (*Bordetella*, *Burkholderia* and *Neisseria*) | KatAB | Stress protein |
| MAG_110 vs pathogenic *Betaproteobacteria* (*Bordetella*, *Burkholderia* and *Neisseria*) | PNAG | Biofilm formation |
| MAG_110 vs pathogenic *Betaproteobacteria* (*Bordetella*, *Burkholderia* and *Neisseria*) | T6SS-II | Secretion system |
| MAG_119 vs pathogenic *Betaproteobacteria* (*Bordetella*, *Burkholderia* and *Neisseria*) | Biotin metabolism | Nutritional virulence |
| MAG_119 vs pathogenic *Betaproteobacteria* (*Bordetella*, *Burkholderia* and *Neisseria*) | Cytochrome c maturation | Iron uptake |
| MAG_119 vs pathogenic *Betaproteobacteria* (*Bordetella*, *Burkholderia* and *Neisseria*) | Hsp60 | Adherence |
| MAG_119 vs pathogenic *Betaproteobacteria* (*Bordetella*, *Burkholderia* and *Neisseria*) | Phospholipases C | Toxin |
| MAG_119 vs pathogenic *Betaproteobacteria* (*Bordetella*, *Burkholderia* and *Neisseria*) | RpoS | Regulation |
| MAG_119 vs pathogenic *Betaproteobacteria* (*Bordetella*, *Burkholderia* and *Neisseria*) | Tap type IV pili | Adherence |
| MAG_119 vs pathogenic *Betaproteobacteria* (*Bordetella*, *Burkholderia* and *Neisseria*) | Type IV pili biosynthesis | Adherence |
| MAG_129 vs pathogenic *Betaproteobacteria* (*Bordetella*, *Burkholderia* and *Neisseria*) | Cytochrome c maturation | Iron uptake |
| MAG_129 vs pathogenic *Betaproteobacteria* (*Bordetella*, *Burkholderia* and *Neisseria*) | icl | metabolic adaptation |
| MAG_129 vs pathogenic *Betaproteobacteria* (*Bordetella*, *Burkholderia* and *Neisseria*) | Tap pili | Adherence |
| MAG_129 vs pathogenic *Betaproteobacteria* (*Bordetella*, *Burkholderia* and *Neisseria*) | Type IV pili biosynthesis | Adherence |
| MAG_172 vs pathogenic *Mycobacterium* | Hsp60 | Adherence |
| MAG_172 vs pathogenic *Mycobacterium* | KatA | Stress protein |
| MAG_172 vs pathogenic *Mycobacterium* | pknG | Secreted proteins |
| MAG_172 vs pathogenic *Mycobacterium* | Pyrimidine biosynthesis | Metabolic adaptation |
| MAG_172 vs pathogenic *Mycobacterium* | Yersiniabactin | Iron uptake |
| MAG_94 vs pathogenic *Tsukamurella* | Methionine sulphoxide reductase | Stress adaptation |
| MAG_94 vs pathogenic *Tsukamurella* | relA | Regulation |
| MAG_12 vs pathogenic *Chlamydia* | Capsule | Immune evasion |
| MAG_12 vs pathogenic *Chlamydia* | Capsule biosynthesis and transport | Colonization and Immune evasion |
| MAG_12 vs pathogenic *Chlamydia* | Catalase | Stress adaptation |
| MAG_12 vs pathogenic *Chlamydia* | Dot/Icm | Secretion system |
| MAG_12 vs pathogenic *Chlamydia* | GPL locus | Cell surface components |
| MAG_12 vs pathogenic *Chlamydia* | Hsp60 | Adherence |
| MAG_12 vs pathogenic *Chlamydia* | LOS | Endotoxin |
| MAG_12 vs pathogenic *Chlamydia* | O-antigen | Unclassified |
| MAG_12 vs pathogenic *Chlamydia* | Pyrimidine biosynthesis | Metabolic adaptation |
| MAG_111 vs pathogenic *Sphingomonas* | AAI/SCI-II T6SS | Secretion system |
| MAG_111 vs pathogenic *Sphingomonas* | Capsular polysaccharide | Antiphagocytosis |
| MAG_111 vs pathogenic *Sphingomonas* | Catalase-peroxidase | Stress adaptation |
| MAG_111 vs pathogenic *Sphingomonas* | ESX-5 | Secretion system |
| MAG_111 vs pathogenic *Sphingomonas* | FarAB | Efflux pump |
| MAG_111 vs pathogenic *Sphingomonas* | Hcp secretion island-1 | Secretion system |
| MAG_111 vs pathogenic *Sphingomonas* | LPS | Endotoxin |
| MAG_111 vs pathogenic *Sphingomonas* | Pantothenate synthesis | Metabolic adaptation |
| MAG_111 vs pathogenic *Sphingomonas* | Phytotoxin phaseolotoxin | Toxin |
| MAG_111 vs pathogenic *Sphingomonas* | polymorphic membrane protein | Adherence |
| MAG_111 vs pathogenic *Sphingomonas* | relA | Regulation |
| MAG_111 vs pathogenic *Sphingomonas* | Sigma A | Regulation |
| MAG_111 vs pathogenic *Sphingomonas* | The repeat in toxin | the repeat in toxin |
| MAG_111 vs pathogenic *Sphingomonas* | Ton system | Iron uptake |
| MAG_111 vs pathogenic *Sphingomonas* | Urease | Acid resistance |
| MAG_39 vs pathogenic *Rhodospirillales* (*Granulibacter, Gluconobacter and Asaia*) | Alginate regulation | Antiphagocytosis |
| MAG_39 vs pathogenic *Rhodospirillales* (*Granulibacter, Gluconobacter and Asaia*) | Capsule | Immune evasion |
| MAG_39 vs pathogenic *Rhodospirillales* (*Granulibacter, Gluconobacter and Asaia*) | Catalase-peroxidase | Stress adaptation |
| MAG_39 vs pathogenic *Rhodospirillales* (*Granulibacter, Gluconobacter and Asaia*) | Cytochrome c maturation | Iron uptake |
| MAG_39 vs pathogenic *Rhodospirillales* (*Granulibacter, Gluconobacter and Asaia*) | Flp type IV pili | Flp type IV pili |
| MAG_39 vs pathogenic *Rhodospirillales* (*Granulibacter, Gluconobacter and Asaia*) | gsp | secretion system |
| MAG_39 vs pathogenic *Rhodospirillales* (*Granulibacter, Gluconobacter and Asaia*) | Mycobactin | Iron uptake |
| MAG_39 vs pathogenic *Rhodospirillales* (*Granulibacter, Gluconobacter and Asaia*) | Nucleoside diphosphate kinase | Phagosome arresting |
| MAG_39 vs pathogenic *Rhodospirillales* (*Granulibacter, Gluconobacter and Asaia*) | O-antigen | Unclassified |
| MAG_39 vs pathogenic *Rhodospirillales* (*Granulibacter, Gluconobacter and Asaia*) | peritrichous flagella | Flagella |
| MAG_39 vs pathogenic *Rhodospirillales* (*Granulibacter, Gluconobacter and Asaia*) | Phytotoxin phaseolotoxin | Toxin |
| MAG_146 vs pathogenic *Rhodospirillales* (*Granulibacter, Gluconobacter and Asaia*) | Capsular polysaccharide | Antiphagocytosis |
| MAG_146 vs pathogenic *Rhodospirillales* (*Granulibacter, Gluconobacter and Asaia*) | Capsule | Immune evasion |
| MAG_146 vs pathogenic *Rhodospirillales* (*Granulibacter, Gluconobacter and Asaia*) | EF-Tu | Adherence and invasion |
| MAG_146 vs pathogenic *Rhodospirillales* (*Granulibacter, Gluconobacter and Asaia*) | EVP | - |
| MAG_146 vs pathogenic *Rhodospirillales* (*Granulibacter, Gluconobacter and Asaia*) | Flp pili | Adherence |
| MAG_146 vs pathogenic *Rhodospirillales* (*Granulibacter, Gluconobacter and Asaia*) | Hcp secretion island-1 | Secretion system |
| MAG_146 vs pathogenic *Rhodospirillales* (*Granulibacter, Gluconobacter and Asaia*) | Imp | Secretion system |
| MAG_146 vs pathogenic *Rhodospirillales* (*Granulibacter, Gluconobacter and Asaia*) | Methionine sulphoxide reductase | Stress adaptation |
| MAG_146 vs pathogenic *Rhodospirillales* (*Granulibacter, Gluconobacter and Asaia*) | PDIM | Cell wall |
| MAG_146 vs pathogenic *Rhodospirillales* (*Granulibacter, Gluconobacter and Asaia*) | peritrichous flagella | Flagella |
| MAG_146 vs pathogenic *Rhodospirillales* (*Granulibacter, Gluconobacter and Asaia*) | Polysaccharide capsule | Secretion system |
| MAG_146 vs pathogenic *Rhodospirillales* (*Granulibacter, Gluconobacter and Asaia*) | RegX3 | Regulation |
| MAG_146 vs pathogenic *Rhodospirillales* (*Granulibacter, Gluconobacter and Asaia*) | SigA | Protease |
| MAG_146 vs pathogenic *Rhodospirillales* (*Granulibacter, Gluconobacter and Asaia*) | T6SS | Secretion system |
| MAG_146 vs pathogenic *Rhodospirillales* (*Granulibacter, Gluconobacter and Asaia*) | T6SS-II | Secretion system |
